# Impacts of maternal microbiota and microbial metabolites on fetal intestine, brain, and placenta

**DOI:** 10.1186/s12915-023-01709-9

**Published:** 2023-10-04

**Authors:** Aleksi Husso, Tiina Pessa-Morikawa, Ville Mikael Koistinen, Olli Kärkkäinen, Hyuk Nam Kwon, Leo Lahti, Antti Iivanainen, Kati Hanhineva, Mikael Niku

**Affiliations:** 1 Department of Veterinary Biosciences, Faculty of Veterinary Medicine, University of Helsinki, Helsinki, Finland; 2 Food Sciences Unit, Department of Life Technologies, University of Turku, Turku, Finland; 3 Institute of Public Health and Clinical Nutrition, School of Medicine, University of Eastern Finland, Kuopio, Finland; 4 Afekta Technologies Ltd., Kuopio, Finland; 5 School of Pharmacy, University of Eastern Finland, Kuopio, Finland; 6 School of Biological Sciences and Basic-Clinical Convergence Research Institute, University of Ulsan, Ulsan, 44610 South Korea; 7 Department of Computing, University of Turku, Turku, Finland

**Keywords:** Host-microbe interactions, Maternal microbiota, Microbial metabolites, Prenatal development, Intestinal immune system, Neural development, Germ-free mice, Non-targeted metabolomics, Transcriptomics, Multi-omics data analysis

## Abstract

**Background:**

The maternal microbiota modulates fetal development, but the mechanisms of these earliest host-microbe interactions are unclear. To investigate the developmental impacts of maternal microbial metabolites, we compared full-term fetuses from germ-free and specific pathogen-free mouse dams by gene expression profiling and non-targeted metabolomics.

**Results:**

In the fetal intestine, critical genes mediating host-microbe interactions, innate immunity, and epithelial barrier were differentially expressed. Interferon and inflammatory signaling genes were downregulated in the intestines and brains of the fetuses from germ-free dams. The expression of genes related to neural system development and function, translation and RNA metabolism, and regulation of energy metabolism were significantly affected. The gene coding for the insulin-degrading enzyme (*Ide*) was most significantly downregulated in all tissues. In the placenta, genes coding for prolactin and other essential regulators of pregnancy were downregulated in germ-free dams. These impacts on gene expression were strongly associated with microbially modulated metabolite concentrations in the fetal tissues. Aryl sulfates and other aryl hydrocarbon receptor ligands, the trimethylated compounds TMAO and 5-AVAB, Glu-Trp and other dipeptides, fatty acid derivatives, and the tRNA nucleobase queuine were among the compounds strongly associated with gene expression differences. A sex difference was observed in the fetal responses to maternal microbial status: more genes were differentially regulated in male fetuses than in females.

**Conclusions:**

The maternal microbiota has a major impact on the developing fetus, with male fetuses potentially more susceptible to microbial modulation. The expression of genes important for the immune system, neurophysiology, translation, and energy metabolism are strongly affected by the maternal microbial status already before birth. These impacts are associated with microbially modulated metabolites. We identified several microbial metabolites which have not been previously observed in this context. Many of the potentially important metabolites remain to be identified.

**Supplementary Information:**

The online version contains supplementary material available at 10.1186/s12915-023-01709-9.

## Background

The development and programming of the immune system, metabolism, and the central nervous system require interactions with the commensal microbiota [1]. Their essential roles are evident in germ-free (GF) mice, which show a multitude of abnormalities in the gastrointestinal tract, intestinal and systemic immunity, metabolism, and even behavior [2, 3]. We are connected with our prokaryotic companions already before birth, as indicated by the few previous studies probing the effects of maternal microbiota on fetal development. The maternal microbiota stimulates the generation of intestinal lymphoid cells in the fetus, and early host-microbe interactions induce tolerance to avoid excessive reactivity and inflammatory pathologies [4–7]. The absence of maternal microbiota during pregnancy predisposes the offspring to metabolic syndrome and affects the differentiation of enteroendocrine cells and the sympathetic nerves [8]. Maternal microbes also promote axonogenesis in the fetal brain and modulate the differentiation of microglia in a sex-specific manner [5, 9, 10].

Live bacteria are rare in a healthy fetus [11, 12]. Thus, the prenatal host-microbe interactions are likely primarily mediated by circulating metabolites and other components of microbes, reaching the fetus through the placenta. Metabolites generated or modified by the gut microbiota penetrate all host tissues [13]. We recently showed that thousands of microbially modulated metabolites are found in the fetus, by non-targeted metabolomics comparison of fetuses from germ-free (GF) and specific pathogen-free (SPF) mouse dams [14]. A hundred compounds were undetectable in the GF animals, indicating that their synthesis is dependent on the microbiota. We identified several metabolites with reported effects on host physiology and fetal development, such as 3‐indolepropionic acid (IPA), trimethylamine N‐oxide (TMAO), and 5‐aminovaleric acid betaine (5-AVAB) [15–17]. Well-studied bacterially derived metabolites include the short-chain fatty acids (SCFAs), produced by microbial metabolism of dietary complex carbohydrates, and microbially modified secondary bile acids [18]. SCFAs contribute to the fetal programming of energy metabolism and sympathetic nervous system development [8]. Experiments with monocolonized dams have revealed the importance of microbial aromatic hydrocarbons on the fetal immune system [6]. The majority of microbial metabolites and especially their effects on mammalian fetal development are however still unknown [1, 14].

Microbial metabolites are sensed by multiple receptor systems, including the SCFA-activated G-protein coupled receptors (GPCRs), nuclear receptors such as aryl hydrocarbon receptor (AhR), pregnane X receptor (PXR), farnesoid X activated receptor (FXR) alias bile acid receptor, and peroxisome proliferator-activated receptors (PPAR) [1, 19–22]. These signaling pathways modulate immunity, energy metabolism, and neurophysiology and are essential in host health and development.

**Fig. 1:**
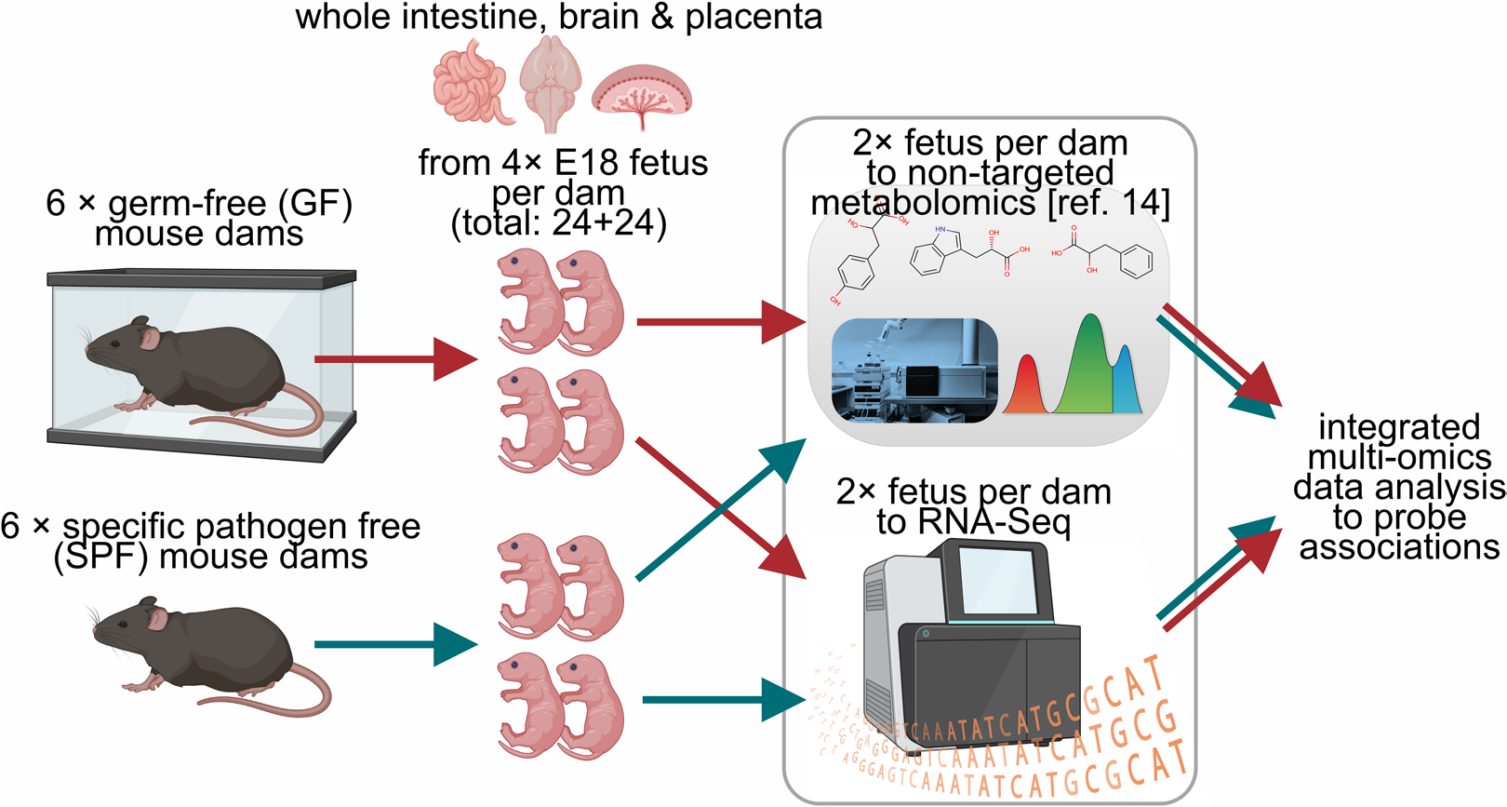
Experimental design. Figure created with Biorender.com

In this study, we investigated the impacts of maternal microbial metabolites on the fetal intestine, brain, and placenta (Fig. 1). We analyzed the gene expression profiles in these organs from fetuses of GF and SPF mouse dams and associated the gene expression data with our recent non-targeted metabolomics data [14, 23]. Our observations indicate major impacts of maternal microbiota on fetal gene expression profiles, strongly associated with microbially modulated metabolites.

## Results

### Fetal intestine

**Fig. 2:**
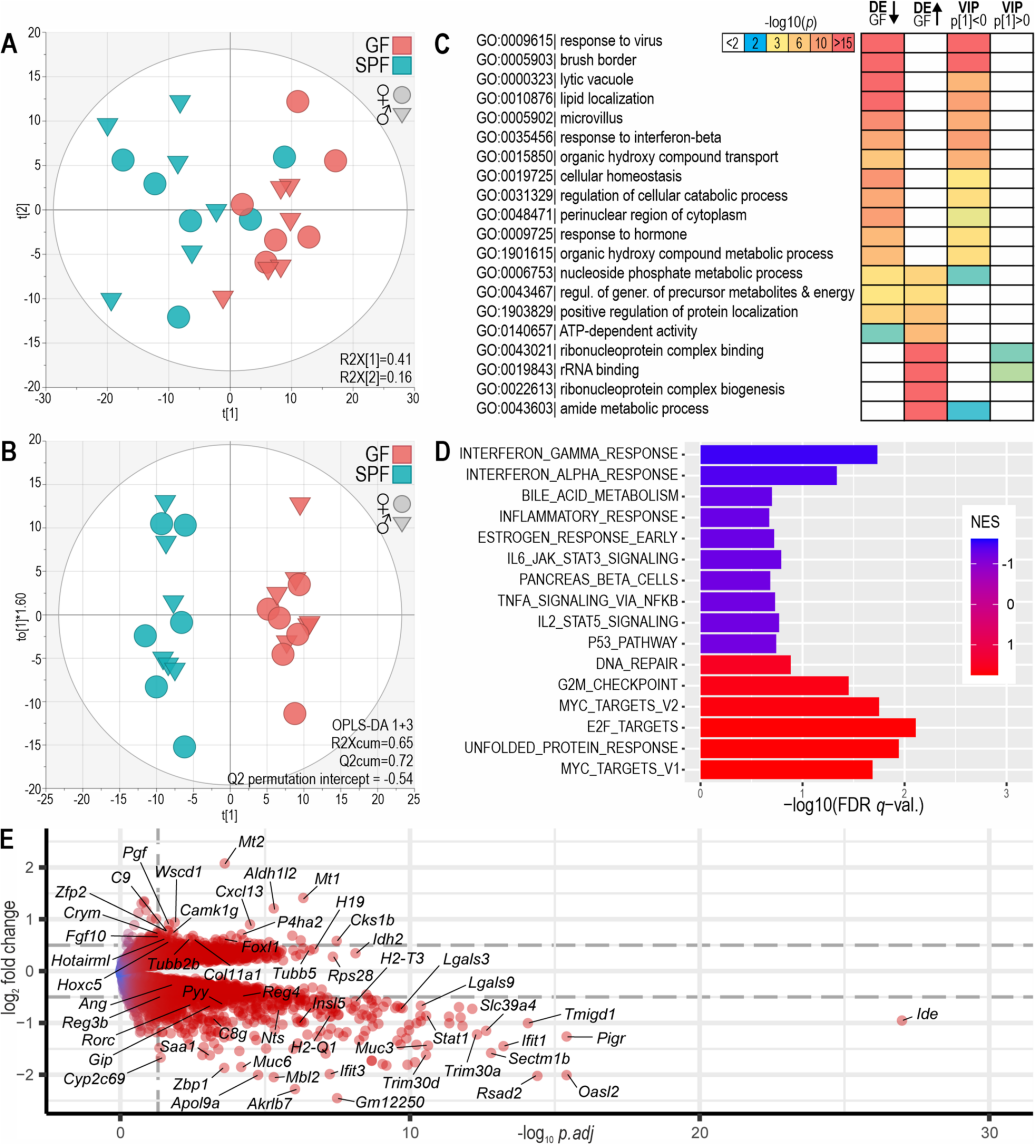
Differential gene expression analysis of GF versus SPF fetal intestine. **A** PCA of 1000 most variable genes. The ellipsoid shows Hotelling’s T2 (95%). **B** OPLS-DA of 1000 most variable genes. **C** ORA of genes which were significantly upregulated or downregulated in GF versus SPF (*p.adj* < 0.05; DE GF up and DE GF down) and of genes with OPLS-DA variable importance in projection (VIP) > 1 and negative or positive S-plot loadings. The top 20 enriched ontology terms are shown; for more details, see Additional file 2: Fig. S1–2. **D** Hallmark gene sets enriched in GSEA. A maximum of 10 of the top gene sets with FDR *q*-value < 25% are shown with negative (blue) and positive (red) net enrichment scores (NES) for GF versus SPF. **E** Volcano plot of differential gene expression. Genes with negative log_2_ fold change values were downregulated in GF fetuses. Dashed lines indicate log_2_ fold change ± 0.5 and adjusted *p* value 0.05

A major impact of maternal microbiota in the fetal intestine was indicated by differential gene expression analysis (Table 1, Additional file 1: Table S1, Fig. 2, and Additional file 2: Fig. S1–S2). GF and SPF expression profiles clustered in principal component analysis (PCA; Fig. 2A). The groups were completely separated in a supervised orthogonal partial least squares discrimination analysis (OPLS-DA, validated by *R*^2^cum and *Q*^2^cum scores and permutation test; Fig. 2B).

**Table 1:** Summary of differential expression analysis of GF vs SPF mouse fetal tissues

Tissue	Genes passing pre-filtering	Downregulated in GF (*p.adj* < 0.05)	Upregulated in GF (*p.adj* < 0.05)
Fetal intestine	13,493	1249 (9.3%)	941 (7.0%)
Fetal brain	14,645	67 (0.46%)	50 (0.34%)
Placenta	13,433	409 (3.0%)	309 (2.3%)

Over-representation analysis (ORA) of differentially expressed (DE) genes (*p.adj* < 0.05) by Gene Ontology (GO) terms suggested an impact of maternal microbiota on the fetal intestinal immune system, epithelial cell physiology, and translation (Fig. 2C; for more details, see Additional file 1: Fig. S1–S2 and Additional file 1: Table S4). The GO categories *response to virus*, *brush border*, *lytic vacuole*, *lipid localization*, *microvillus*, and *response to interferon beta* were most strongly over-represented among genes downregulated in the GF intestine (Fig. 2C). *Amide metabolic process*, *ribonucleoprotein complex biogenesis and binding*, and *rRNA binding* were over-represented among genes upregulated in GF. Over-representation analysis (ORA) of genes with high variable importance in projection (VIP) in OPLS-DA showed similar enrichment.

Gene set enrichment analysis (GSEA) using the hallmark gene sets indicated downregulation of the *interferon alpha* and *interferon gamma* response gene sets in the GF fetal intestine (NES < 0, FDR < 5%; Fig. 2D, Additional file 1: Table S5a). The leading-edge genes included *Irf7*, *Irf9*, and *Stat* transcription factors; *B2m* (a component of the MHC complex); and interferon-stimulated genes (ISGs) such as *Rsad2*, *Ifi44*, and *Oasl* (Additional file 2: Fig. S5). Negative enrichment scores of gene sets related to interferon signaling and virus response were also seen in the GF fetal intestine in the C2 curated pathways and C7 immunosignature gene sets (Additional file 1: Table S5b). Proliferation-associated gene sets (such as *Myc targets*, *E2F targets*, *G2M checkpoint*) and *unfolded protein response* were upregulated in GF versus SPF fetuses (NES > 0; Fig. 2D). The hallmark gene sets do not cover all of the GO categories enriched in ORA.

A higher number of genes was downregulated than upregulated in the GF fetal intestine (Table 1, Fig. 2E). These were enriched for immunity-related gene sets in ORA (Fig. 2 and Additional file 2: Fig. S2). Strikingly, 27 out of the 30 genes most strongly downregulated in the GF fetal intestine were involved in immunity, intestinal host-microbe interactions, and xenobiotic metabolism (*p.adj* < 0.05 and ranked by fold change). The genes significantly downregulated in GF fetuses included mucins, interferon and cytokine signaling genes such as *Stat1-3*; virus response genes such as *Trim* and *Oasl* families and *Rsad2*; interleukins and their receptors (such as *Il18*, *Il18R*, *Il34*, and *Il10rb*); the major acute phase response gene *Saa1*; several complement genes; 7 MHC-I genes (but none of the MHC-II genes); antimicrobial lectins (*Reg3b*, *Reg4*, *Lgals3*, *Lgals4*, *Lgals8*, *Lgals9*); the antimicrobial peptide *Ang*; and the marker of commensal microbiota associated regulatory T cells and ILC3 cells *Rorc*. Tight junction component (*Cldn15*, *Cldn19*, *Tjp3*) and enteroendocrine cell marker genes *Insl5*, *Pyy*, *Gip*, and *Nts* were also downregulated. However, the expression of interferon genes was not detectable in the fetal intestine. In contrast, 80% of the genes annotated for translation, ribosomes, and tRNA metabolism were upregulated in the GF fetal intestine, including several subunits of the translation initiation factor eIF2 and the elongator acetyltransferase complex. The genes most strongly upregulated in the GF fetal intestine included metallothioneins *Mt1* and *Mt2*, the B cell chemoattractant *Cxcl13*, developmental regulators *Foxl1* and *Hoxc5*, collagen biosynthesis genes *P4ha2* and *Col11a1*, and various genes involved in metabolism.

Regarding the predicted transcription factor binding sites, the DE genes in the intestine (and in the brain and placenta) were most significantly enriched for E2Fs, ZF5, and FOXN4 (Additional file 1: Table S4). Predicted targets of interferon regulatory factors (IRFs) and early growth response proteins (EGRs) were also significantly enriched. DE genes in the fetal intestine (but not in the brain and placenta) were significantly enriched for multiple predicted binding sites of AhR and AhR nuclear translocator (Arnt). DE genes in the intestine and placenta (but not in the brain) were also significantly enriched for binding sites for the vitamin D receptor (VDR) and/or FXR.

### Fetal brain

**Fig. 3:**
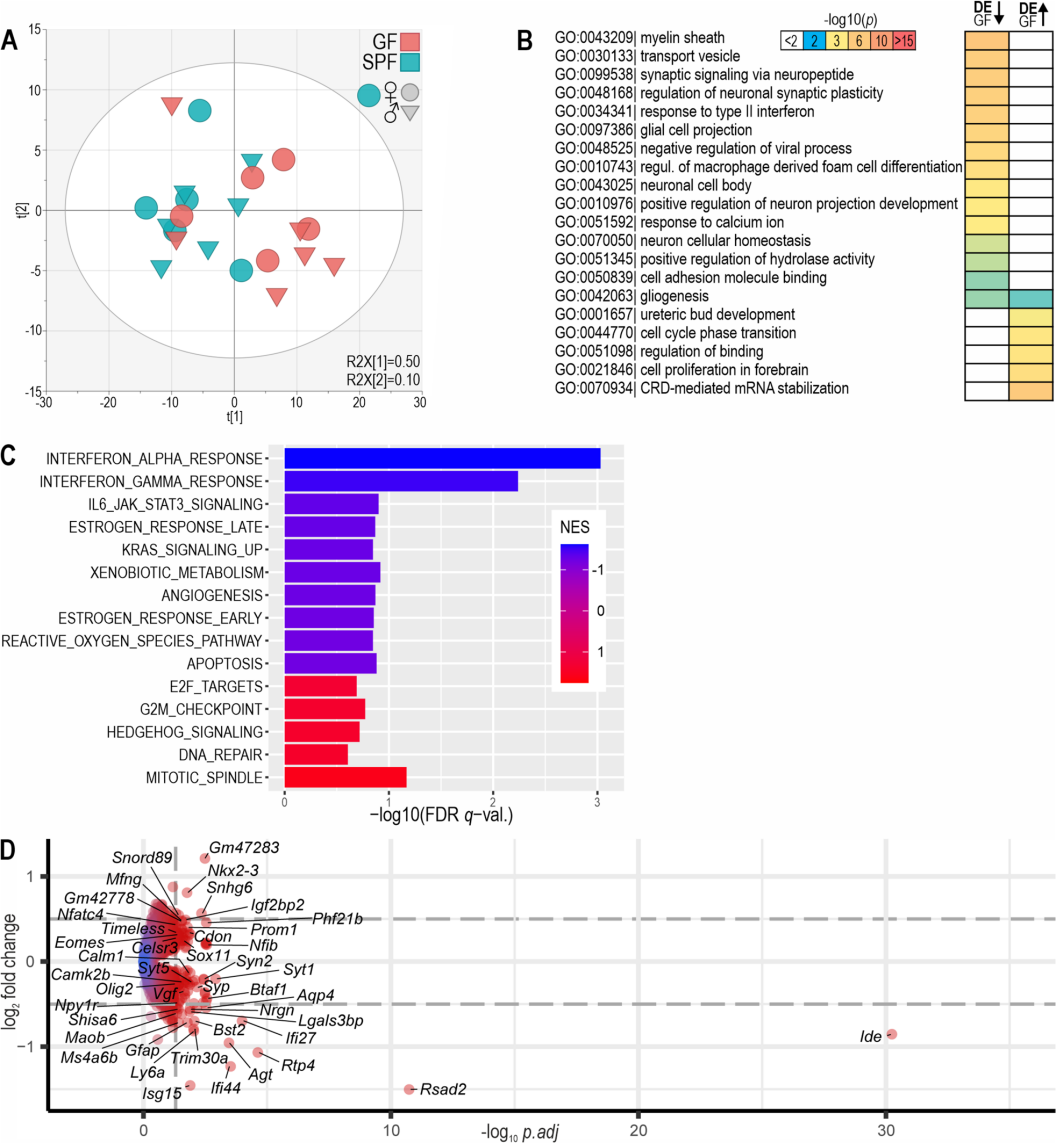
Differential gene expression analysis of GF versus SPF fetal brain. **A** PCA of 1000 most variable genes. The ellipsoid shows Hotelling’s T2 (95%). **B** ORA of genes which were significantly upregulated or downregulated in GF versus SPF (*p.adj* < 0.05; DE GF up and DE GF down). The top 20 enriched ontology terms are shown; for more details, see Additional file 2: Fig. S1, S3. **C** Hallmark gene sets enriched in GSEA. A maximum of 10 of the top gene sets with FDR *q*-value < 25% are shown with negative (blue) and positive (red) net enrichment scores (NES) for GF versus SPF. **D** Volcano plot of differential gene expression. Genes with negative log_2_ fold change values were downregulated in GF fetuses. Dashed lines indicate log_2_ fold change ± 0.5 and adjusted *p* value 0.05

In the fetal brain, differences in gene expression profiles were clearly less prominent. GF and SPF fetuses were not discriminated by PCA (Fig. 3A), and no valid OPLS-DA model separating the groups was obtained (best model: 1 + 7 components; *R*^2^Xcum 0.80, *Q*^2^cum 0.05, *Q*^2^ permutation intercept −0.27). ORA indicated significant enrichment in GO categories related to neural functions (such as *myelin sheath*, *glial cell projection*, *modulation of chemical synapse*, and *axon*) as well as antiviral immunity among genes downregulated in the GF fetal brain (Fig. 3B and Additional file 2: Figs. S1, S3). Some neural development categories were upregulated in GF fetuses.

Among the hallmark gene sets with negative enrichment scores in GSEA for the fetal GF brain, the most significant were *interferon alpha* and *interferon gamma* response sets (Fig. 3C). The *mitotic spindle* gene set was most positively enriched in GF versus SPF mice (Fig. 3C).

Also in the brain, the genes downregulated in GF fetuses were enriched for immunity-related genes (Additional file 1: Table S4). The most strongly differentially expressed genes included interferon and virus response genes such as *Rsad2*, *Ifi44*, *Ifi27*, *Rtp4*, *Trim30a*, and *Lgals3bp*; the glymphatic aquaporin *Aqp4*; and the lymphocyte antigen *Ly6a* (Fig. 3D). The expression of interferon genes was not detectable. Multiple genes involved in neuronal development and synaptic signaling were significantly differentially expressed. *Nrgn*, two synaptotagmins, *Shisa6*, *Calm1*, the monoamine oxidase gene *Maob*, and the glial-specific *Gfap* and *Olig2* were significantly downregulated. Genes upregulated in the GF brain included the neural stem cell regulator *Phf21b*; the neural transcription factors and activators *Nfib*, *Sox11*, and *Eomes*; several cadherins involved in neural system development; and the lncRNAs *Snhg6* and *Gm47283*.

### Placenta

**Fig. 4:**
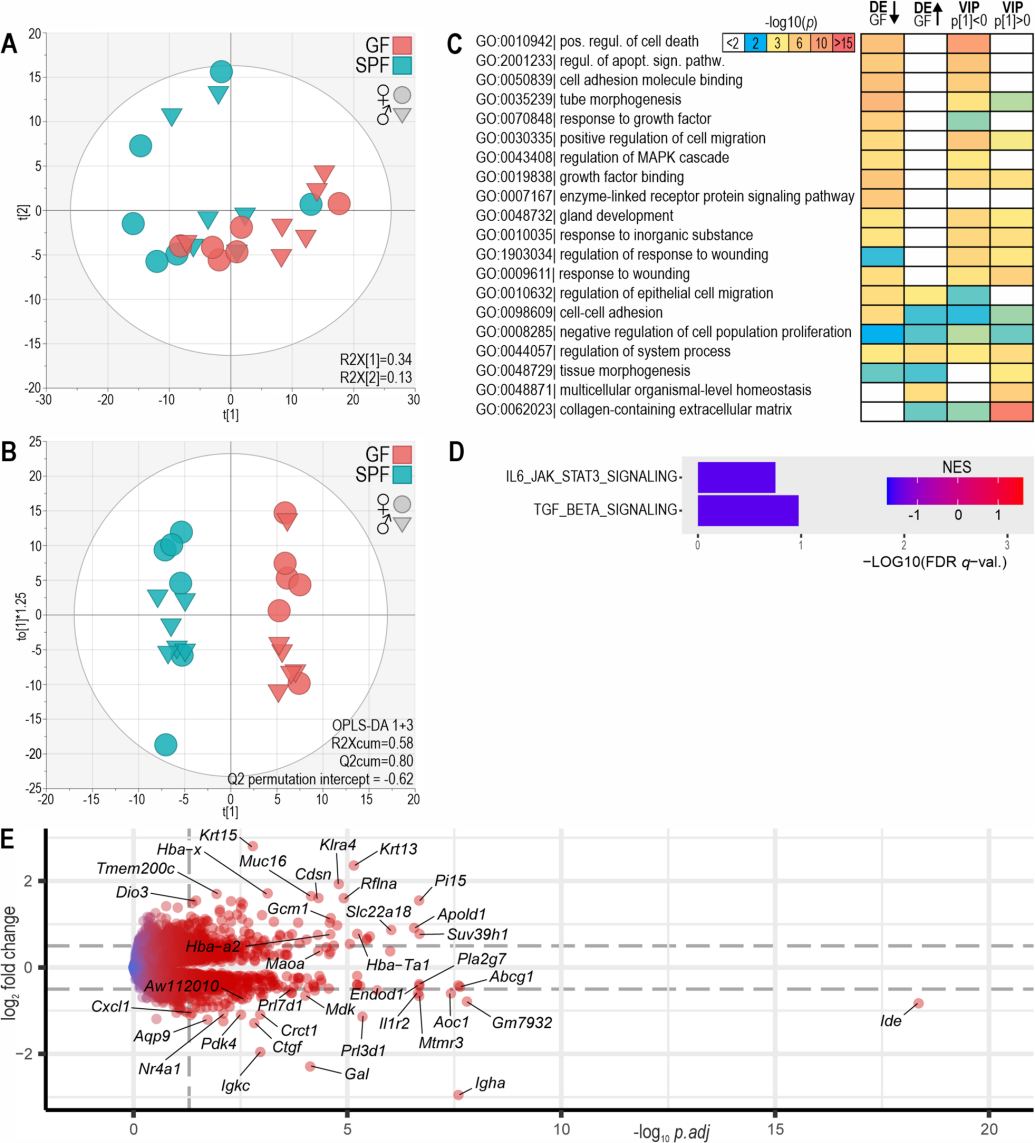
Differential gene expression analysis of GF versus SPF placenta. **A** PCA of 1000 most variable genes. The ellipsoid shows Hotelling’s T2 (95%). **B** OPLS-DA of 1000 most variable genes. **C** ORA of genes which were significantly upregulated or downregulated in GF versus SPF (*p.adj* < 0.05; DE GF up and DE GF down) and of genes with OPLS-DA variable importance in projection (VIP) > 1 and negative or positive S-plot loadings. The top 20 enriched ontology terms are shown; for more details, see Additional file 2: Figs. S1, S4. **D** Hallmark gene sets enriched in GSEA. The gene sets with FDR *q*-value < 25% are shown. **E** Volcano plot of differential gene expression. Genes with negative log_2_ fold change values were downregulated in the GF placenta. Dashed lines indicate log_2_ fold change ± 0.5 and adjusted *p* value 0.05

In the placenta, GF and SPF gene expression profiles were not separated in PCA but could be discriminated by a validated OPLS-DA model (Fig. 4A, B). ORA indicated differences in gene sets related to development, tissue homeostasis, and immunity. GO categories *positive regulation of cell death*, *regulation of apoptotic signaling pathway*, *cell adhesion molecule binding*, and *tube morphogenesis* were most significantly enriched among genes downregulated in the GF placenta; *collagen-containing extracellular matrix* and *multicellular organismal-level homeostasis* were enriched among upregulated genes (Fig. 4C; Additional file 2: Figs. S1, S4).

In GSEA, only two hallmark gene sets were negatively enriched in the GF placenta (*IL6-JAK-STAT-signaling* and *TGF-beta-signaling*; Fig. 4D). No significantly enriched hallmark gene sets in GF over SPF mice were detected in the placenta.

The genes most strongly downregulated in the GF placenta (Fig. 4E) included immunoglobulin and complement genes, the recently characterized bacterial response gene *AW112010*, the interleukin receptor *Il1r2* implicated in endometriosis, the diamine oxidase *Aoc1* implicated in pregnancy regulation, the prolactin precursor *Prl3d1*, the exosomal endonuclease *Endod1*, the neuroendocrine peptide *Gal*, the mitogen *Mdk*, the mitochondrial pyruvate dehydrogenase kinase *Pdk4*, and the lncRNAs *Gm7932* and *4933417E11Rik*. The expression of the interferon genes *Ifnk* and *Ifne* was detectable in the placenta, but it was not statistically significantly stronger in the SPF animals. A majority (23 out of 30) of the most strongly differentially expressed genes were upregulated in the GF placenta. The most upregulated genes included the placental gene regulator *Gcm1*, the trypsin inhibitor *Pi15*, the killer cell receptor *Klra4*, typical epithelial and endothelial genes, hemoglobins, and solute carriers.

### Differentially expressed genes common to the fetal intestine, brain, and placenta

Only seven genes were observed to be significantly differentially expressed in all three tissues: the insulin-degrading enzyme *Ide*; the virus response genes *Rtp4*, *Rsad2*, and *Isg15*; the transcription regulator *Btaf1*; the Rho GTPase *Rhou*; and the tubulin *Tuba4a*. A total of 26 genes were significantly differentially expressed both in the fetal intestine and brain, and 129 genes were shared between the intestine and placenta. All these gene sets shared between the tissues were enriched for GO terms for immunity, virus response, and symbiont interaction (not shown). However, more specific GOs (< 500 genes) were largely different.

### Sex differences

The DE analysis comparing GF versus SPF fetuses was controlled for sex. When GF and SPF fetuses were compared in each sex separately, male fetuses appeared more sensitive to modulation by maternal microbial status. In the intestine, 1223 genes were significantly differentially expressed in male GF versus SPF fetuses, while only 307 genes were significantly DE in female GF versus SPF fetuses. In the brain, 8 genes were DE in males versus 2 genes in females, and in the placenta, 145 versus 36 genes. The total numbers of significantly DE genes were smaller in these analyses due to the smaller comparison groups. Genes which were significantly DE in GF versus SPF in the male intestine or placenta but not in females were enriched for development, biosynthesis, and histone methylation gene sets (not shown).

We also compared the gene expression in all male fetuses versus female fetuses, controlling for the differences in GF versus SPF animals (Additional file 1: Table S3). In the fetal brain, all the significantly DE genes were sex chromosomal. In the intestine, several autosomal interferon-inducible genes were significantly upregulated in the male fetuses in comparison with females. In the placenta, 57 autosomal genes were significantly DE in male versus female fetuses; these were enriched for development-related genes but not immunity by ORA.

### Associations between gene expression profiles and metabolite abundancies

To explore the potential effects of microbial metabolites on the fetal intestine, brain, and placenta, we analyzed the associations between gene expression and metabolites. We utilized our previously published metabolomics data [14, 23] and focused on metabolites which were undetectable in GF fetuses or significantly less abundant than in SPF fetuses. In total, 2200 molecular features were included in the analysis, being significantly more abundant in SPF fetuses in at least one tissue. Ninety-nine of these were only detected in SPF mice. To detect various types of associations between individual metabolites and genes, and between molecular feature groups and gene families or co-regulated pathways, we evaluated (1) correlations of abundances of individual metabolites with expression levels of individual genes, (2) correlations of clusters of metabolites with clusters of genes (by hierarchical clustering), and (3) correlations within biclusters composed of metabolites and genes.

We focused on metabolites and genes which showed significant differences between the experimental groups and strong correlations also within the SPF group (Spearman *ρ* > 0.9), thus representing metabolite-gene associations which indicate dose–response and are likely due to actual abundances of the compounds, rather than due to the overall differences of GF/SPF physiology. As the small size of the fetal organs required gene expression and metabolite profiling to be done from different fetuses, the association analysis was performed using the averages of two fetuses from each dam. All twin pairs were highly similar in terms of gene expression profiles (Spearman *ρ* range = 0.969–0.994) and metabolite profiles (*ρ* = 0.926–0.991) and significantly more similar than fetuses from different dams (transcriptomes: *ρ* = 0.897–0.995, significantly lower at *p* < 0.001; metabolomes: *ρ* = 0.655–0.981, *p* < 0.001). Thus, examining the associations calculated across littermates is justified. The approach emphasizes associations to maternally derived metabolites, as the exposure to those is expected to be similar in the fetuses from the same litter.

**Fig. 5:**
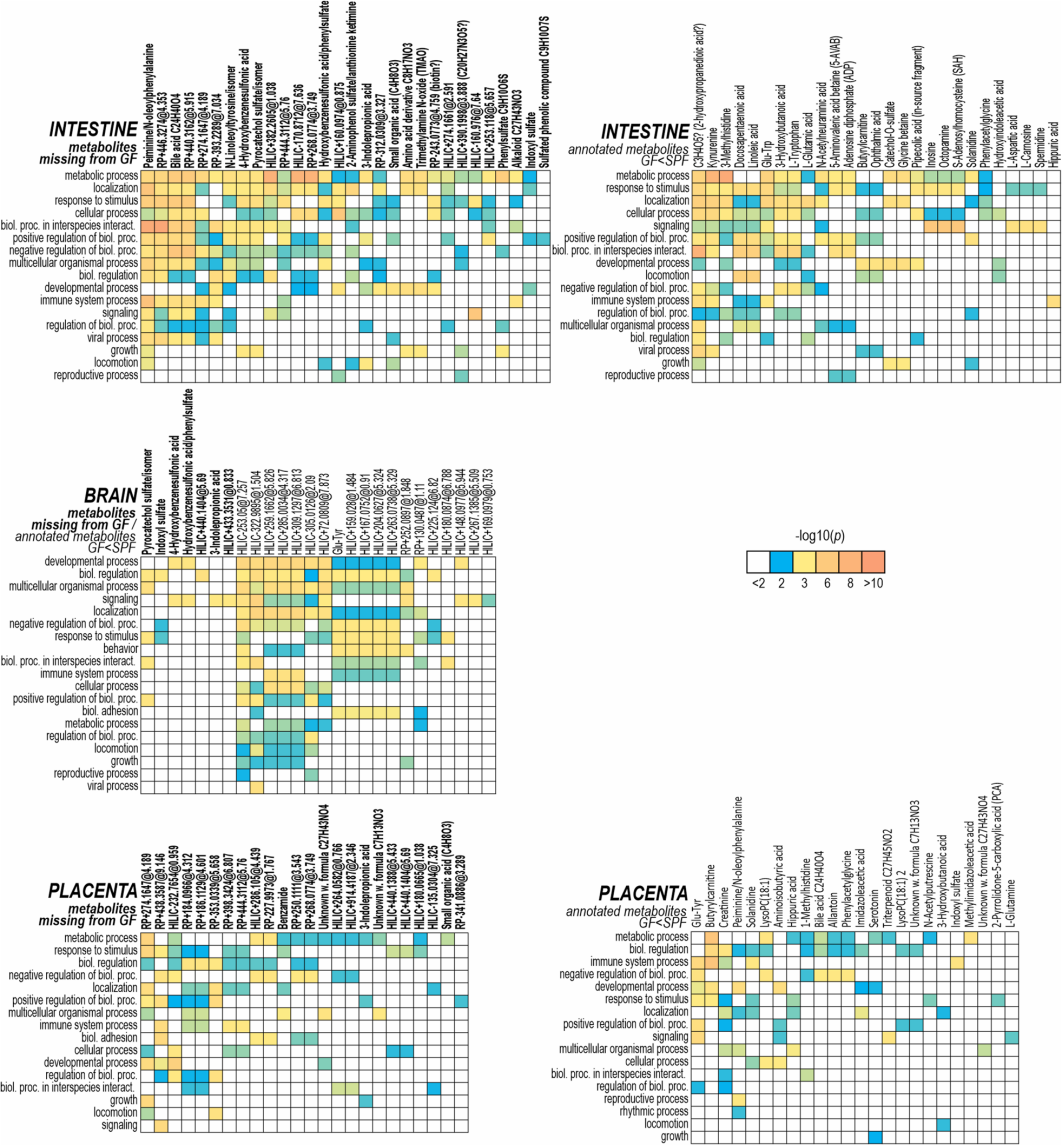
Over-representation analysis of genes strongly associated with metabolites in the fetal intestine, brain, and placenta. Highest-scoring metabolites missing from GF fetuses (in bold text) and highest-scoring annotated metabolites more abundant in SPF fetuses (in regular text) are shown. R, RP column; H, HILIC column (positive / negative)

All molecular features were scored based on significant hits in ORA of the strongly associated genes or gene clusters (Additional file 1: Table S4). Highest-scoring molecular features were then examined in more detail by ORA of genes which were strongly directly associated with them (Fig. 5; for more detail, see Additional file 2: Figs. S6–S8). An overview of the metabolite-gene associations is shown in Additional file 1: Table S7. In the intestine and placenta, the expression levels of immunity genes (by GO annotations) were mostly positively correlated with metabolomics signal intensities. In contrast, genes associated with translation correlated mostly negatively with metabolites in the fetal intestine. In the brain, such differences were not observed, but genes with GO annotations for immunity or neurophysiology had more metabolite associations.

**Fig. 6:**
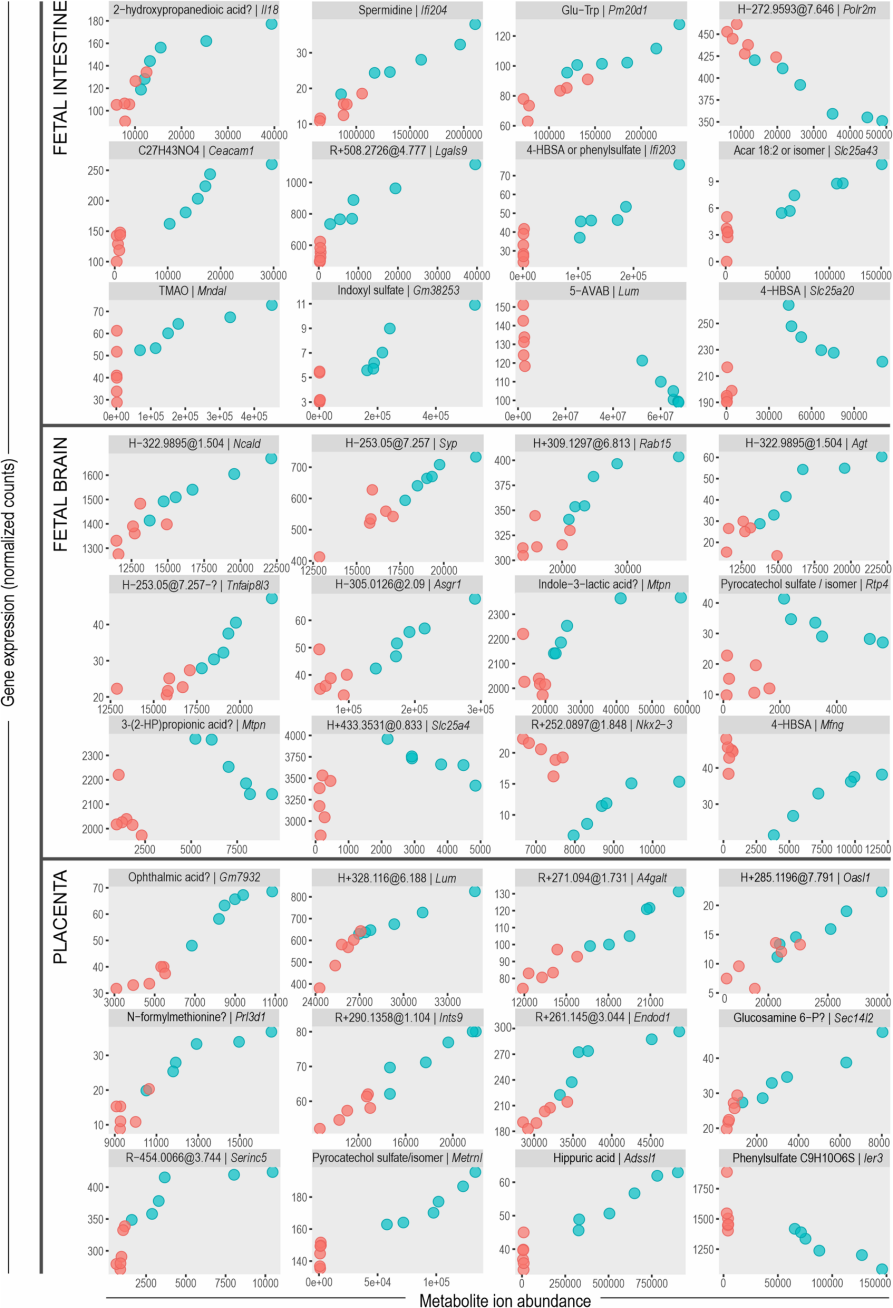
Examples of associations between metabolites and gene expression in the fetal intestine, brain, and placenta. Only one gene per metabolite is shown. Metabolite signal intensity (ion abundance) on the *X*-axis and gene expression (normalized counts) on the *Y*-axis. Each data point is the mean of the two fetuses from one dam. Red = GF; turquoise = SPF. Tentatively annotated metabolites are marked with “?”. R, RP column; H, HILIC column (positive/negative)

Examples of associations between individual molecular features and genes are shown as scatterplots in Fig. 6.

A total of 148 metabolites which were not detectable in the GF fetal intestine or brain showed associations with gene expression profiles (Additional file 1: Table S6). Eighty-four of these were also missing from the GF placenta. Twenty-three of the molecular features missing from GF fetuses were at least putatively characterized. Six of these were aryl sulfates (4-hydroxybenzenesulfonic acid, indoxyl sulfate, pyrocatechol sulfate, and phenyl sulfates). The aryl sulfates were among the metabolites with the strongest associations to the gene expression patterns in all tissues investigated. In the ORA of the fetal intestine, they were associated with immunity (response to biotic stimulus and activation of innate immune response), lipid metabolism, regulation of cell growth, aromatic compound biosynthesis, and tRNA metabolism. In the brain, aryl sulfates were associated with viral infection pathways, dopaminergic synapse, Ras signaling, and response to organocyclic compounds. In the placenta, they did not show significant associations.

3-Indolepropionic acid (IPA) and two unknown compounds (all observed only in SPF fetuses) were associated with the adaptive immune system and Ras signal transduction in the brain. In the fetal intestine, IPA was primarily associated with RNA metabolism, and in the placenta, with the regulation of cell growth.

Kynurenine, tryptophan, and 3-methylhistidine were associated with immunity (largely positively) and RNA metabolism (mostly negatively) in the intestine. 1-Methylhistidine was associated with virus response in the placenta. Several other amino acids and their derivatives were also significantly more abundant in the SPF fetuses.

Aryl sulfates and tryptophan derivatives are typical AhR ligands. As fetal intestinal DE genes were enriched for predicted AhR/Arnt targets, we specifically looked at associations of such annotated metabolites with transcription factor binding sites. Genes strongly associated with tryptophan, kynurenine, IPA, a phenyl sulfate, and several metabolites with tentative annotations as tryptamine and indoleacetic acids were significantly enriched for predicted AhR/Arnt binding sites. This was not observed for indoxyl sulfate, hydroxyindoleacetic acid, 4-hydroxybenzenesulfonic acid, and pyrocatechol sulfates. Instead, indoxyl sulfate, 4-hydroxybenzenesulfonic acid, pyrocatechol sulfates, and metabolites tentatively annotated as indoleacetic acids were associated with predicted VDR targets.

The betaine trimethylamine N-oxide (TMAO; only detected in the SPF fetuses) was associated with brush border, absorption, and lipid metabolism in the SPF fetal intestine. In the brain, the only strong correlation (positive) was with the *Ide* gene. In the placenta, there were no significant ORA hits for genes strongly associated with TMAO. 5-AVAB was associated with response to biotic stimulus and lipid metabolism in the intestine.

Butyrylcarnitine and several unannotated molecular features were associated with immunity (response to biotic stimulus, virus response, neutrophil degranulation, interleukin-1 production; including the *Rorc* gene) and lipid metabolism in the intestine. Also, in the placenta, butyrylcarnitine was associated with neutrophil degranulation and with oxidoreductase and peroxidase activities.

A bile acid (with retention time matching the secondary bile acid chenodeoxycholic acid), N-linoleyltyrosine or its isomer, and peiminine or N-oleoylphenylalanine (putative classifications) and two unannotated features were strongly associated with multiple pathways in the intestine. These included immunity (response to biotic stimulus, innate immunity, pattern recognition receptor signaling, T lymphocyte differentiation, several virus response pathways), translation, brush border, and intestinal absorption. These compounds showed no significant ORA hits in the brain or placenta.

The dipeptides Glu-Trp and Glu-Tyr were associated with extracellular response stimulus, virus responses, and other immunity pathways in all tissues investigated.

The largest number of direct associations with genes (306 genes with Spearman *ρ* > 0.9 in SPF mouse intestine data) were observed for an unidentified metabolite with the probable formula C3H4O5 (possibly 2-hydroxypropanedioic acid alias tartronic acid), which was not detected in the GF intestine but was present in brain and placenta in both experimental groups. In ORA, for the fetal intestine, the associated genes were significantly enriched for immunity pathways (response to virus, cytokine signaling, and adaptive immune response).

Several unidentified compounds were associated with virus response, translation, and ribonucleoprotein pathways in the intestine. One of these (RP + 278.1254@1.34) matched the MS1 mass of queuine. This compound was undetectable in the GF intestine and placenta and significantly less abundant in the GF brain. It was associated with genes primarily enriched for translation and its initiation, ribosomes (including the aminoacyl-tRNA binding protein Rpl8), protein-l-isoaspartate (d-aspartate) O-methyltransferase activity, nonsense-mediated decay, and E2F-1 targets in the intestine. In the brain, there were multiple unannotated molecular features which were more abundant in SPF fetuses and associated with neural development, synaptic function, and interferon/virus responses. In the placenta, several unidentified compounds were associated with immune responses (inflammatory response, cytokine production, and phagocytosis).

Some genes showed unexpected nonlinear dependencies on metabolites. For example, *Slc25a20* was inversely correlated with 4-hydroxybenzenesulfonic acid in the SPF fetal intestine, but the gene was downregulated in the GF intestine, where this metabolite was undetectable (Fig. 6; last plot for intestine). This pattern was most commonly observed for aryl sulfates, trimethylated compounds, and other metabolites not detected in GF fetuses. It was more common in the brain than in the intestine or placenta. In the fetal intestine, genes with such nonlinear dependencies were enriched for negative regulation of innate immune response, lipid metabolism, and negative regulation of Notch signaling (not shown). Genes with linear correlations were enriched for positive regulation of defense response, complement activation, and translation. This was not observed in the brain or placenta.

## Discussion

Our analysis of the fetal intestine, brain, and placenta from GF and SPF murine dams reveals major impacts of maternal microbiota on mammalian prenatal gene expression, associated with circulating microbial metabolites. Gene expression profiling indicated a profound effect of the maternal microbial status on genes critical for the fetal immune system, translation, metabolism, and neurophysiology. The effects were strongest in the fetal intestine. We observed multiple metabolites of likely microbial origin which were strongly associated with these processes in the fetus. Several of these metabolites have not been previously characterized in this context.

### Fetal intestine

In the fetal intestine, critical genes for host-microbe interactions, innate immunity, and epithelial barrier were affected by the maternal microbial status. Genes coding for mucins and the polymeric immunoglobulin receptor were among the most significantly downregulated in the GF fetuses. Expression of *Rorc*, the gene for a transcription factor critical for commensal microbiota tolerance, was also significantly suppressed, as were the genes for several antimicrobial peptides and lectins. Inflammation, interferon responses, and interleukin signaling pathways were broadly downregulated in GF fetuses at the level of gene expression.

Exploring the associations of the differentially expressed genes with metabolites on the level of gene ontology, we found that various known metabolites of potential microbial origin were broadly associated with immunity. These metabolites include aryl sulfates such as 4-hydroxybenzenesulfonic acid and indoxyl sulfate, as well as the amino acid tryptophan and its derivatives kynurenine and 3-indolepropionic acid (IPA). The dipeptides Glu-Trp and Glu-Tyr were also strongly associated with immunity genes. The endogenous L-Glu-L-Trp and especially the bacterially synthesized γ-D-Glu-L-Trp have been investigated as immunomodulatory compounds which stimulate interferon signaling [24]. These different isomers could not be distinguished in our LC–MS analysis.

SCFA and secondary bile acids are well-known mediators of host-microbe interactions [18]. SCFAs were not optimally detected by our metabolomics pipeline. However, the carnitine conjugate of butyrate was among the metabolites most significantly associated with immunity in the intestine. A bile acid matching chenodeoxycholic acid was also strongly associated with immunity.

Some of the microbially modulated metabolites with strong associations with gene expression could not yet be annotated. A molecular feature with the probable formula C3H4O5 (possibly 2-hydroxypropanedioic acid, also known as tartronic acid or hydroxymalonate) showed the largest number of associated genes, enriched for immunity pathways.

Several mucin genes (and the associated trefoil family factors) were downregulated in our GF murine fetuses. Their expression showed strong correlations with metabolites present in lower levels in the GF mice, such as kynurenine, 5-AVAB, and the unidentified C3H4O5. Secreted and membrane-bound mucins form the interface for host-microbe interactions in the intestine [25]. The structure of the mucin layers is modulated by gut microbiota [26, 27], but mucin expression begins early in fetal development [28–30]. Previous studies in adult mice have yielded conflicting results, with GF mice showing either downregulation [31] or upregulation [32] of mucins when compared to microbially colonized animals.

We observed significant downregulation of the polymeric immunoglobulin receptor (pIgR) gene in the GF fetal intestine. Glu-Trp dipeptide was among the identified metabolites that correlated with its expression. In addition, strong correlations were found for several unidentified compounds. pIgR transports IgA across the intestinal epithelium to the apical surface and is cleaved to generate the secretory component [33]. In adult mice, pIgR expression is modulated by gut microbiota, possibly by TLR- and MyD88-mediated signaling. It is however expressed already early in fetal development in intestinal and other epithelia [34].

RORγt (a splice variant of the *Rorc* gene) is a marker for intestinal regulatory T cells (Tregs) and group 3 innate lymphoid cells (ILC3s) which both mediate tolerance to commensal microbiota [35, 36]. *Rorc* was downregulated in the GF fetal intestine, accompanied by downregulation of *Stat3*, a known mediator of microbiota signaling in ILCs and intestinal epithelium [37]. This suggests that commensal tolerance may begin to be established already before birth. Butyryl carnitine and ophthalmic acid were among the metabolites with associations to *Rorc* gene expression.

The antimicrobial peptide angiogenin [38] and C-type lectins Reg4 [39] and Reg3b [40] regulate gut microbiota composition by selectively inhibiting, e.g., α-proteobacteria, *Enterobacteriaceae*, or gram-negative bacteria, respectively. Reg3b is induced by the microbial metabolite propionate [40]. Several metabolites, including indoxyl sulfate correlated strongly with the expression of these genes.

The differentially expressed interferon (IFN) response genes included IFN signal transduction mediators, notably *Irf7*, *Irf9*, and *Stat2* which are key factors in transcriptional activation of IFN downstream effectors [41], and numerous interferon-stimulated genes (ISGs) such as *Rsad2*, *Ifi44*, several *Trim*s and *Oasl*s which exhibit various types of anti-viral effector or regulatory activities [42, 43]. High representation of ISGs among differentially expressed genes has been reported in GF compared to conventional piglets [44]. *Ifi44*, *Ifit1*, and *Rsad2* found in our study were among the genes with lower expression in GF piglet tissues, including the intestine.

The downregulation of virus response pathways in GF animals may be due to presumably lower exposure to viruses or indirectly due to the lack of bacterial immune activation. GF or antibiotic-treated mice are more susceptible to viral infections than conventional mice, and antibiotics induced a downregulation of interferon response genes in mouse pups [45, 46]. Gut microbes are known to modulate the host response to systemic viruses, such as the influenza virus [47, 48]. This may be due to tonic signals provided by the microbiota that set the homeostatic levels of the immune system components, including type 1 IFN, and calibrate the type 1 IFN response during viral infection. Potential mechanisms include activation of cells of the immune system through engagement of the pattern-recognition receptors (PRR) by binding of microbial molecules like lipopolysaccharides (LPS) [48]. Recent observations suggest a role for small-molecular microbial metabolites, such as secondary bile acids [49]. We found several metabolites with associations with interferon response. Most of these remained unknown; tryptamine and ethyl-3-indoleacetic acid were putatively identified.

The expression of the actual interferon genes was not detected in the fetal intestine or brain. This suggests that the downregulation of interferon response genes in the fetus may be an indirect effect due to lower interferon levels in the GF dams, although we only measured gene expression in two fetal organs. To our knowledge, the placental transfer of interferons has not been investigated in the mouse. Interferons and most cytokines are thought not to cross the human placenta, with permeability similar to the murine placenta [50–52]. The placenta is an important contributor to antiviral immunity in pregnancy [53], and we detected the expression of two interferon genes in the murine placenta. In contrast to interferons, several interleukin genes were strongly expressed, and some were significantly downregulated in the GF fetal intestine.

Our observations complement and extend previous research on the prenatal effects of maternal microbiota on intestinal immunity and permeability [6]. In that study, monocolonization of GF murine dams only during pregnancy impacted the offspring’s postnatal intestinal expression of > 190 genes common to our study. In another study, antibiotic treatment of neonatal mice induced significant changes in the distal small intestinal expression of > 240 genes common to our study, almost exclusively in the same direction [45]. In contrast to the study by Gomez de Aguero et al., these also included multiple interferon signaling genes common to our data, all downregulated in the antibiotic-treated pups [6].

Many of the metabolites with correlations to immune gene expression are aromatic hydrocarbons and known ligands for AhR. Fetal exposure to maternal microbiota and bacterial AhR ligands is necessary for the differentiation of intestinal group 3 innate lymphoid cells and balanced intestinal immunity [6, 54]. Several AhR ligands were also strongly associated with genes enriched for predicted binding sites of AhR [55] or VDR and for known target genes of these and other xenobiotic-sensing nuclear receptors like PXR [56]. However, some of the canonical AhR target genes (such as those coding the cytochrome P450 family 1 enzymes) were not significantly differentially expressed in GF and SPF fetuses. This suggests that the effects of these compounds in the fetus may be mediated by non-canonical AhR signaling or other xenobiotic signaling pathways.

Associations of microbially modulated metabolites with immunity-related genes were mostly positive. However, aryl sulfates, trimethylated compounds, bile acids, and certain other metabolites showed mostly negative correlations with immunity genes, which was surprising considering that many of these compounds are generally considered proinflammatory. In GF fetuses, they were associated with unexpected nonlinear gene expression profiles: when gene expression was negatively correlated with metabolite signal in SPF fetuses, the GF fetuses showed *lower* expression than SPF fetuses for most of these genes; for positively correlating genes, the GF expression levels were *higher*. These genes were enriched for *negative* regulation of immunity (whereas genes with expected associations in GF fetuses were enriched for *positive* regulation). Our observations may suggest that these microbial metabolites downregulate anti-inflammatory genes; in GF fetuses, these pathways may be inactive in the absence of microbial proinflammatory signals.

The maternal microbial status also had a major impact on genes mediating fetal metabolism. In the intestine, immunity and lipid metabolism are intimately connected. Mice lacking adaptive immunity have lower expression of key lipid transporters such as CD36 [37]; most of these were also significantly downregulated in the GF fetal intestine. TMAO, 5-AVAB, and several microbially derived metabolites with associations with immunity pathways, such as aryl sulfates, butyryl carnitine, and chenodeoxycholic acid had associations with lipid metabolism in the intestine. TMAO, and its bacterially produced precursor TMA have been widely implicated in metabolic pathologies including atherosclerosis, metabolic disease, human type 2 diabetes, and gestational diabetes [57]. Multiple biological mechanisms of action have been demonstrated in human and mouse models [57, 58], with the molecular roles of these compounds strongly dependent on the experimental setting and context. 5-AVAB can inhibit beta oxidation of fatty acids [16], which may be pertinent for its associations with lipid metabolism.

*Ide* (coding for insulin-degrading enzyme) was by far the most downregulated gene in the GF intestine and in the other tissues. To our knowledge, this effect of the maternal microbiota has not been previously reported in the fetus, but downregulation of *Ide* was recently observed in adult GF or antibiotic-treated mice on a high-fat diet [59]. The GF dams probably had lower levels of circulating insulin [60]. Maternal insulin is thought not to cross the placenta, but it does stimulate placental nutrient transport [61]. Thus, the differential *Ide* expression in the fetus may be an indirect consequence of higher nutrient availability, which likely affects fetal insulin levels. However, *Ide* expression was also strongly and positively associated with the levels of several microbially modulated metabolites (including IPA in the intestine and TMAO in the brain), suggesting that it may be more directly modulated in the fetus by the maternal microbiota.

Interestingly, several *Ceacam* genes were also downregulated in our GF intestine (some of which were also observed by de Aguero et al. or by Garcia et al. [6, 45]). Ceacam1 is involved in both insulin signaling [62], tolerogenic immunological signaling [63], and mucosal colonization [64] and may therefore connect these processes [59].

Translation and RNA metabolism genes were upregulated in the GF fetal intestine. Aryl sulfates, tryptophan, and its derivatives (including IPA) were among the metabolites associated with these pathways. Gene expression profiling did not indicate an obvious rationale for an increased protein synthesis, although mitosis and stress response pathways were over-represented among genes upregulated in the GF intestine [65]. In the adult GF intestine, proliferation is suppressed, and the characteristic enlargement of the caecum is secondary to mucus and fiber accumulation. The upregulation of the components of the translation machinery may be a compensatory response to the depletion of queuine, a hypermodified nucleobase exclusively synthesized by bacteria but essential to tRNA stability and function in eukaryotes [66]. Depletion of modified tRNAs is associated with pauses, misincorporation of amino acids, and +1 frame shifts during translation, leading to protein misfolding and aggregation [67]. Queuine was recently implicated in intestinal inflammation and permeability [68]. Interestingly, GSEA strongly indicated upregulation of unfolded protein response genes in the GF intestine, and the *Qtrt1* gene coding for queuine tRNA-ribosyltransferase catalytic subunit was slightly upregulated. We detected a metabolite matching the MS1 mass of queuine, which was undetectable in the GF intestine and placenta and significantly less abundant in the GF brain and was associated with the expression of genes for translation, ribosomes, protein repair, and nonsense-mediated decay. Many of the genes for proteins affected by queuosine depletion in cell culture [69] and multiple genes linked to human tRNA modopathies [70] were significantly differentially expressed in the GF fetal intestine. These observations suggest that due to the lack of gut microbiota, the GF dam may be unable to provide sufficient queuine for the fetus, which then utilizes all available queuine (originating from the dam diet) in queuosine synthesis.

### Fetal brain

In the fetal brain, gene expression analysis indicated a substantial impact of maternal microbial effects on genes related to neurodevelopment and brain antiviral immunity. The effects were much more limited than in the intestine. This is likely due to the blood–brain barrier restricting the entry of microbial metabolites [14, 15, 71], although it should be noted that the blood–brain barrier permeability appears to be increased in GF mice [72].

Numerous genes belonging to the interferon alpha and gamma pathways had significantly lower expression in the GF fetal brain, including *Rsad2*, *Ifi44*, *Ifi27*, *Rtp4*, *Trim30a*, and *Bst2*. On the wider Gene Ontology level, immunity was correlated with metabolites including aryl sulfates, benzenesulfonic acids, betaines, acylcarnitine, hippuric acid, and pyridoxamine. Pyrocatechol sulfate and benzenesulfonic acids were among the metabolites with correlations to genes belonging to interferon pathways.

*Rsad2*, also known as viperin, was the most significantly differentially expressed gene in the brain after *Ide*. Its expression was also significantly lower in the GF fetal intestine and placenta. Rsad2 protein expression is induced by interferons, and it has a major role in the antiviral defense of the cell [73]. It catalyzes the production of ddhCTP which is a direct inhibitor of virus replication as a chain terminator of RNA-dependent RNA polymerases. It may also have other mechanisms of antiviral action [74]. The importance of gut microbiota on brain resistance to virus infection was recently shown by Yang et al.: depletion of gut microbiota in mice exacerbated the neurological symptoms of encephalomyocarditis virus infection concurrently with diminished innate immune responses and decreased expression of ISGs [75].

Maternal microbiota appears to have a major impact on fetal neurodevelopment and may also promote neurodevelopmental abnormalities in the context of maternal inflammation [76]. Neurobehavioral impacts of perinatal antibiotic exposure were recently reported [77]. Genes coding for multiple key neuronal and glial transcription factors and other regulators, structural proteins, and synaptic signaling components were differentially expressed in our GF and SPF fetuses, with almost all synaptic genes (by Gene Ontology annotations) downregulated in the GF fetal brain. Aryl sulfates, betaines, hippuric acid, and several amino acids correlated with genes belonging to neuronal gene ontologies.

Modulation of fetal neurodevelopment by maternal microbiome likely involving microbial metabolites such as TMAO, hippuric acid, and 5-AVAB, has been reported [10]. However, the differentially expressed genes were almost completely different from those reported in our study, primarily affecting axonogenesis and not enriched for any type of immunity genes (not shown). Vuong et al. studied younger (E14.5) fetuses, suggesting that the maternal microbiota modulates fetal neurodevelopment at several different stages. The lack of maternal microbiota affected the microglia in E18.5 mice, especially in male fetuses [9]; differential expression of *Rtp4*, *Ifi27*, *Ifitm3*, and *Bst2* were observed also here. Immunity and neurodevelopment may be linked by the cytokine CX3CL1 (fractalkine, neurotactin), which was significantly downregulated in our GF fetuses [78]. In the brain, the CX3CL1 receptor (CX3CR1) is exclusively expressed in the microglia. In CX3CR1-deficient mice, synaptic pruning and signaling and brain connectivity are deficient, leading to disruption in social interaction and behavior [79].

### Placenta

In the placenta, several critical regulators of pregnancy were differentially expressed: genes coding for prolactin and the histamine-degrading diamine oxidase Aoc1 [80] were downregulated in GF mice, whereas the trypsin inhibitor Pi15 and the transcription factor Gcm1 were upregulated. The maternal microbiota broadly modulated genes associated with growth and morphogenesis, especially blood vessel development, and immunity. Aryl sulfates, imidazoles (e.g., creatinine and imidazoleacetic acid) and indoles including IPA and 5-hydroxyindoleacetic acid were among the metabolites with wide overall associations with differentially expressed genes in the placenta. The strongly downregulated lncRNA gene *AW112010* was recently shown to also code an LPS-responsive protein which apparently coordinates mucosal innate responses [81]; the lncRNA Gm7932 was recently implicated in virus defense response [82]. Interestingly, *Endod1*, coding for an exosomal protein, was among the genes most significantly downregulated by maternal microbiota. Extracellular vesicles are thought to mediate immunological interactions in the placenta, and it is tempting to speculate that their production could be modulated by the maternal microbiota [83].

### Sex differences

Maternal microbial status affected the gene expression in male fetuses more widely than in female fetuses. The genes which were differentially regulated in GF versus SPF fetuses only in males were mostly related to development and biosynthesis. Disregarding the maternal microbial status, the sexual dimorphism of gene expression profiles was strongest in the placenta. Several interferon response genes were upregulated in the male fetal intestine compared to the female intestine. These findings are unexpected, as generally stronger immune responses are observed in adult females [84]. Interestingly, a similar developmental stage-specific sex dimorphism was reported for murine microglial responses to microbiota: male fetuses but female adults were more strongly affected [9]. Our observation suggests that this may be a more systemic characteristic of male fetuses, which may contribute to their greater susceptibility to prenatal complications [85].

### Limitations of the study

This is an explorative study; in vitro and in vivo experiments with purified compounds identified here will be necessary to show the actual causal effects of microbial metabolites on gene expression and development. Meaningful statistical significances could not be computed for the observed associations between metabolites and gene expression. Over the whole dataset (including both GF and SPF animals), statistical significance is to be expected, as we examine the associations of differentially expressed genes and differentially abundant metabolites. Within the SPF group, the observed associations were largely not statistically significant, due to the large number of metabolomics signals and genes and the limited number of data points. However, we only considered multiple very strong correlations (Spearman *ρ* > 0.9) generating highly significant hits in the over-representation analysis; we believe this to be a reliable approach to screen for metabolites which are most likely to be physiologically relevant. Many of the chemically identified compounds prioritized by this method are well-known immunomodulators.

Some of the differences in GF versus SPF fetuses may be due to the different metabolism and immune system of the pregnant GF dams, rather than the direct effects of maternal microbial metabolites on the fetal tissues [3, 86]. These cannot be segregated in mammals as the dam, its microbiota, the fetus, and the placenta as an active interface are intimately interconnected; ultimately, the causative factor is the maternal microbial status. The physiological differences of GF dams do not explain the reported associations of microbial metabolites and gene expression profiles, as we focused on associations which could be observed within the SPF group. The associations of predicted targets of xenobiotic receptors with known ligands suggest direct modulation of fetal gene expression by microbial metabolites. Some of the most obvious systemic mediators (maternal interferons and insulin) are probably not effectively transferred through the placenta [50–52, 61].

Gene expression profiling obviously does not always reflect protein levels and actual physiology. On the other hand, some of the potential impacts of maternal microbiota may not be captured, such as possible modulation of the antigen receptor repertoires of the adaptive immune system which requires macromolecular ligands.

Many of the metabolites potentially originating from the microbial metabolism are often considered harmful. Indoxyl sulfate and hippuric acid and the indoles kynurenine and indoleacetic acid are well-known uremic toxins in the context of kidney diseases [87], contributing to inflammation, cardiovascular diseases, and metabolic and hormonal and neuronal dysfunction. Animals evolved under constant exposure to microbial metabolites, and the developing fetus is also expected to tolerate or even require these compounds. Evaluating the physiological consequences of fetal microbial metabolite exposure and immune activation mediated by these compounds requires studies in postnatal animals. It is also context-dependent: while GF animals are obviously abnormal in some respects, other implications of the deficits in early host-microbe interactions are only realized in the face of environmental stressors [46]. We are therefore studying the early host-microbe interactions in large production animals, in addition to laboratory mice living in strictly controlled conditions [88].

## Conclusions

Maternal microbiota has a major impact on fetal gene expression. Genes essential for the immune system, neurophysiology, translation, and energy metabolism are strongly affected already before birth. The impact is especially pronounced in the fetal intestine. Also, in the developing brain, the impact of microbial metabolites appears substantial, although less profound, possibly due to the blood–brain barrier which limits the metabolite exposure of the neural tissue. Male fetuses are more broadly affected by maternal microbiota than female fetuses.

The gene expression differences are associated with microbially modulated metabolites. Aryl sulfates and other AhR ligands; the trimethylated compounds TMAO and 5-AVAB; Glu-Trp, and other dipeptides; fatty acid derivatives; and the tRNA nucleobase queuine are among the metabolites strongly associated with fetal gene expression. Many of the potentially important microbial metabolites remain to be identified.

## Methods

### Animals

Fetal and placental mouse organ samples from pregnant GF (*n* = 6) and SPF (*n* = 6) C57BL/6 J dams were obtained from the EMMA Axenic Service at Instituto Gulbenkian de Ciência, Portugal, as described in detail previously [14]. The dams were euthanized 18.5 days post-coitum. The whole brain, whole intestine, and whole placenta were collected from 4 fetuses per dam (two for gene expression profiling and two for metabolomics), for a total of 12 fetuses per experimental group per method. The fetal organ samples were frozen in liquid nitrogen immediately after collection, stored at –80 °C, and shipped on dry ice. The GF and SPF statuses of the dams were regularly monitored by culture and 16S RNA gene qPCR. The GF dams were 3–4 months old, and the SPF dams were 4–5 months old. All dams were fed identical RM3-A-P breeding diets (SDS Special Diet Services, Essex, UK), autoclaved at 121 °C. The SPF feed was autoclaved for 20 min and the GF feed for 30 min due to logistical reasons.

### RNA extraction and gene expression profiling

Total RNA was extracted from the whole fetal intestine, whole fetal brain, and whole placenta (one female fetus and one male fetus per dam) using the Qiagen RNeasy Mini Kit (Qiagen, Germany). The tissues were mechanically lysed by grinding with a plastic pestle (Bel-Art, PA) attached to an electric drill, and the protocol included the optional on-column DNAse digestion.

The quality and quantity of the extracted RNA samples were analyzed with LabChip GX Touch HT RNA Assay Reagent Kit (PerkinElmer, Waltham, MA, USA) and Qubit RNA BR kit (Thermo Fisher Scientific, Waltham, MA, USA). Genomic DNA contamination was measured using the Qubit DNA BR kit. The intestinal RNA extracts were re-purified using the Qiagen RNeasy Micro Plus kit to remove the remaining genomic DNA; the brain and placental extracts did not require additional purification.

Dual-indexed mRNA libraries were prepared from 150 ng of total RNA with QuantSeq 3′ mRNA-Seq Library Prep Kit FWD (Lexogen Gmbh, Vienna, Austria) according to the user guide version 015UG009V0251. During the second strand synthesis, 6-bp unique molecular identifiers (UMI) were introduced with the UMI Second Strand Synthesis Module (Lexogen Gmbh, Vienna, Austria) for the detection and removal of PCR duplicates. The quality of the libraries was measured with LabChip GX Touch HT DNA High Sensitivity Reagent Kit (PerkinElmer, Waltham, MA, USA). Sequencing was performed with NovaSeq 6000 System (Illumina, San Diego, CA, USA) with a read length of 2 × 101 bp and a target coverage of 10 M reads for each library.

The QuantSeq 3′ mRNA-Seq Integrated Data Analysis Pipeline version 2.3.1 FWD UMI (Lexogen Gmbh, Vienna, Austria) on Bluebee® Genomics Platform (Illumina, CA) was used for the primary quality evaluation of the RNA sequencing data, removing PCR duplicates by the unique molecular identifier (UMI) sequences, alignment by STAR Aligner [89] with modified ENCODE settings, gene read counting by HTSeq-count [90] with QuantSeq FWD-specific options, and DESeq2 [91] for DE analysis.

DE analysis comparing GF versus SPF fetuses and controlling for sex was performed using DESeq2 version 1.38.3, for each tissue type separately. Genes were pre-filtered by requiring ≥ 5 reads in ≥ 12 fetuses (the number of fetuses in one group).

One brain sample was excluded from all analyses as an outlier, as expression profiling identified it as other head tissue. Therefore, brain data was prefiltered for ≥ 5 reads in ≥ 11 fetuses.

### Metabolomics

Metabolites were analyzed from the whole fetal intestine, whole fetal brain, and whole placenta (one female fetus and one male fetus per dam; with the exception of one dam in both experimental groups providing two male fetuses, due to availability). The metabolomics method has been published previously [14]. Briefly, the samples were analyzed by a liquid chromatography–mass spectrometry (LC–MS) system, consisting of a 1290 Infinity Binary UPLC coupled with a 6540 UHD Accurate-Mass Q-TOF (Agilent Technologies Inc., Santa Clara, CA, USA), as described previously [14]. Briefly, a Zorbax Eclipse XDB-C18 column (2.1 × 100 mm, 1.8 μm; Agilent Technologies) was used for the reversed-phase (RP) separation and an Acquity UPLC BEH amide column (Waters Corporation, Milford, MA, USA) for the HILIC separation. The peak detection and alignment were performed as previously reported and described in Klåvus et al. by Afekta Technologies Ltd. (Kuopio, Finland) [92]. The metabolite annotations, focusing on molecular features missing from the GF mice (and thus likely representing microbial metabolites) were performed in MS-DIAL v4.70 [93] based on in-house and publicly available spectral databases and in MS-FINDER v3.50 [94] using in silico molecular formula and MS/MS fragmentation prediction.

Putative annotations were now obtained for 17 previously unannotated molecular features which were missing from GF fetuses [14]. We used the standard metabolite identification levels: 3 = putatively annotated compound class, 2 = putatively annotated compound, and 1 = confidently identified compound.

### Statistical analysis

Multiple hypothesis-adjusted *p*-values for DE analyses were calculated using the Benjamini–Hochberg correction. Genes with *p.adj* < 0.05 were considered significantly differentially expressed.

Multivariate statistical analysis including principal component analysis (PCA) and orthogonal partial least squares discrimination analysis (OPLS-DA) was done using SIMCA version 17.0 (Umetrics, Umeå, Sweden). Data were first transformed in DESeq2 using regularized logarithm (rlog) and pareto scaled in SIMCA.

Over-representation analyses (ORA) were performed using Metascape 3.5 [95] with gene prioritization by evidence counting and selective GO clusters, and g:profiler version e109_eg56_p17_1d3191d [96]. Transcription factor binding sites were analyzed using Transfac [97] through g:profiler.

Gene set enrichment assay (GSEA) by functional class scoring [98] was performed with the GSEA version 4.3.2 using mouse-ortholog hallmark gene sets (50 gene sets) from Mouse Molecular Signatures Database Collection v2023.1 and curated (6495 gene sets) and immunosignature gene sets (5219 gene sets) from Human Molecular Signatures Database Collection v2023.1. The expression datasets contained 18,196 genes after collapsing the features to gene symbols. Limits for the number of genes in queries were set at min 15 and max 500 and phenotype permutations at 1000.

Volcano plots were generated using the R package *EnhancedVolcano* version 1.18.0 [99].

Associations between metabolomics and transcriptomics data (as averages of the two fetuses per dam) were explored in R version 4.1.2, using *phyloseq* version 1.38 [100] and *microbiome* version 1.17.2 [101]. Associations of individual molecular features and genes were evaluated by Spearman correlations, selecting pairs which showed *ρ* > 0.9 in SPF mouse data for further analyses.

Hierarchical clustering was performed using the R core method *hclust* (complete linkage), using the dissimilarity matrix (1-Spearman). Associations between hierarchical gene and molecular feature clusters were evaluated by the mean Spearman correlations of cluster members, and clusters with the mean *ρ* > 0.7 in the SPF group (for brain, *ρ* > 0.6, due to the much smaller number of DE genes) were selected for further analyses. These contained, as averages per tissue, 16–43 genes or 7–15 molecular features per cluster.

Plaid model biclustering was performed using *biclust* version 2.0.3 [102]. The biclusters were generated with fit.model = *y* ~ *m* from correlation matrix [abs(Spearman)] using the SPF mouse data only. Approximately ten biclusters were generated for each tissue. The biclusters with the strongest mean internal correlations between molecular features and genes were selected for further analyses.

Molecular features were scored based on the order of numbers of significant hits in g:profiler over-representation analyses (ORA) for their associated gene sets (combined score based on direct correlations, hierarchical cluster correlations, and biclustering).

The top-scoring molecular features in each tissue were selected for ORA of the strongly associated genes (Spearman *ρ* > 0.9 in SPF mouse data). Heatmaps were generated using Metascape 3.5 with gene prioritization by evidence counting, selective GO clusters, all available murine terminologies, and protein–protein interaction enrichment.

The R code is available at Zenodo (https://doi.org/10.5281/zenodo.7267763).

### Supplementary Information

**Additional file 1: Table S1.** Differential gene expression analysis and normalized counts in fetal intestine, brain and placenta: GF vs SPF. **Table S2.** Raw counts. **Table S3.** Differential gene expression analysis in fetal intestine, brain and placenta: male vs female. **Table S4.** g:Profiler over-representation analysis of significantly differentially expressed genes in fetal intestine, brain and placenta. **Table S5.** Gene set enrichment analysis: Mouse MSigDB Hallmark gene sets, Human C2 curated gene sets and Human C7 immunologic signature gene sets in fetal intestine, brain and placenta. **Table S6.** List of metabolites, with prioritization scores. **Table S7.** Overview of metabolite-gene associations.

**Additional file 2: Fig. S1.** Over-represention analysis of genes which were significantly diffrentially expressed in GF versus SPF fetal murine intestine, brain and placenta. **Figs. S2-S4.** Over-representation analyses of downregulated and upregulated genes. **Fig. S5.** Gene set enrichment analysis: heatmaps for interferon alpha and gamma response gene sets. **Figs. S6-S8.** Over-representation analyses of genes strongly associated with metabolites.

## Data Availability

The metabolomics dataset [14] can be accessed at EUDAT (https://doi.org/10.23728/b2share.4be0ea9f87b84a06be960d6a1c4b0b42) [23]. The RNASeq data is in ArrayExpress (accession E-MTAB-12442) [103]. The R code used in the data analysis is available at Zenodo (https://doi.org/10.5281/zenodo.7267763) [104].

## Abbreviations

5-AVAB: 5‐Aminovaleric acid betaine
AhR: Aryl hydrocarbon receptor
DE: Differential gene expression
EGR: Early growth response protein
FXR: Farnesoid X activated receptor/bile acid receptor
GF: Germ-free
GSEA: Gene set enrichment analysis
GO: Gene Ontology
IFN: Interferon
IPA: 3-indolepropionic acid
IRF: Interferon regulatory factor
ISG: Interferon-stimulated gene
LPS: Lipopolysaccharide
OPLS-DA: Orthogonal partial least squares – discrimination analysis
ORA: Over-representation analysis
PCA: Principal component analysis
pIgR: Polymeric immunoglobulin receptor
PXR: Pregnane X receptor
SCFA: Short-chain fatty acid
SPF: Specific pathogen-free
TMA: Trimethylamine
TMAO: Trimethylamine N‐oxide
UMI: Unique molecular identifier
VDR: Vitamin D receptor
