# Supplementary material for: Impacts of maternal microbiota and microbial metabolites on fetal intestine, brain, and placenta
Source: BMC Biol. 2023 Oct 4;21:207. doi: 10.1186/s12915-023-01709-9 (PMC10552303; doi:10.1186/s12915-023-01709-9)
Supplement: Supplementary file 2 — Additional file 2: Fig. S1. Over-represention analysis of genes which were significantly diffrentially expressed in GF versus SPF fetal murine intestine, brain and placenta. Figs. S2-S4. Over-representation analyses of downregulated and upregulated genes. Fig. S5. Gene set enrichment analysis: heatmaps for interferon alpha and gamma response gene sets. Figs. S6-S8. Over-representation analyses of genes strongly associated with metabolites. [file 12915_2023_1709_MOESM2_ESM.pdf]

Supplementary Figures

Husso et al. 2023 Impacts of maternal microbiota and microbial metabolites on fetal intestine, brain and placenta

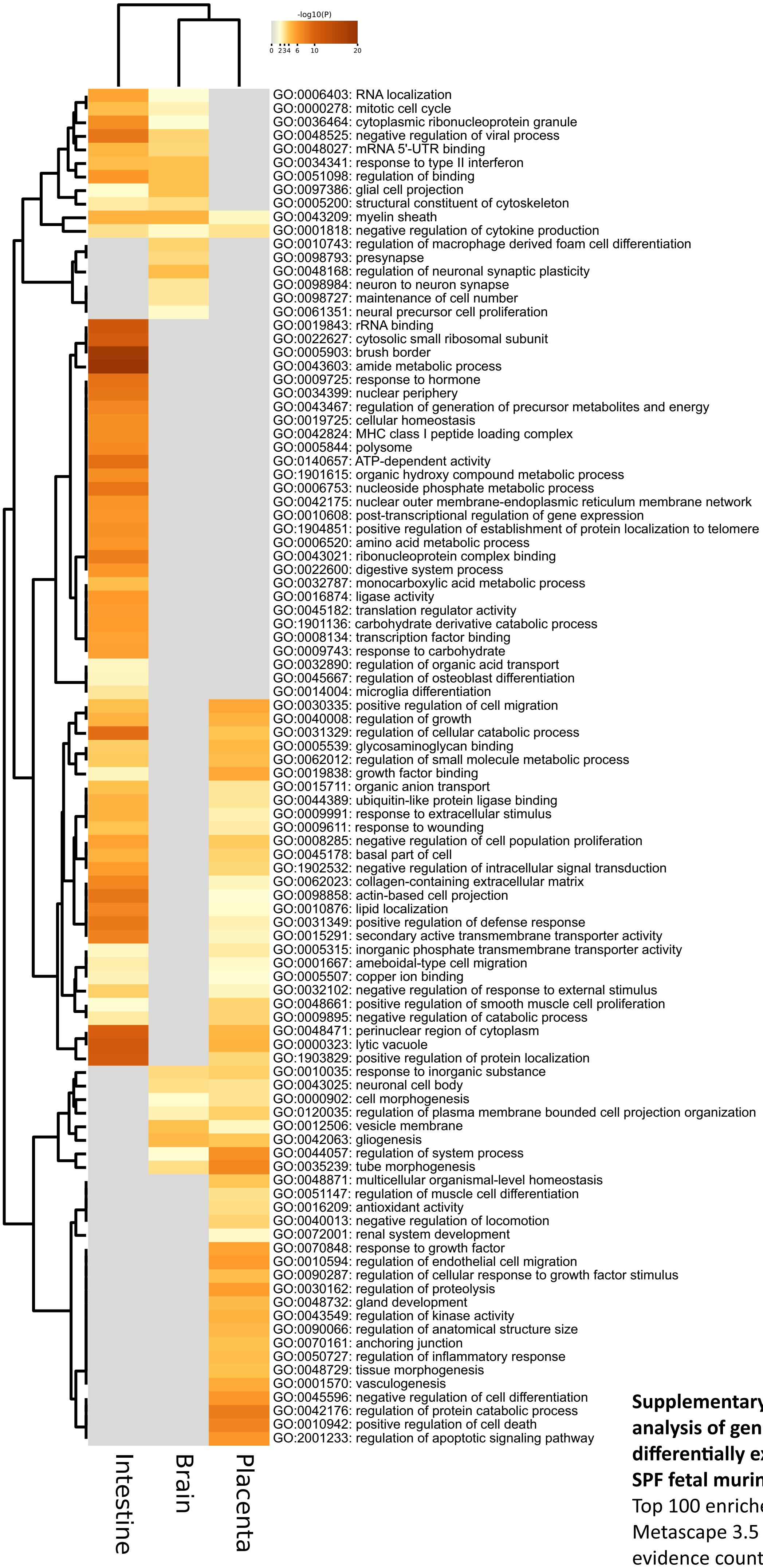

Supplementary Figure 1. Over-representation analysis of genes which were significantly differentially expressed (q < 0.05) in GF versus SPF fetal murine intestine, brain and placenta. Top 100 enriched ontology terms are shown. Metascape 3.5 using gene prioritization by evidence counting and selective GO clusters.

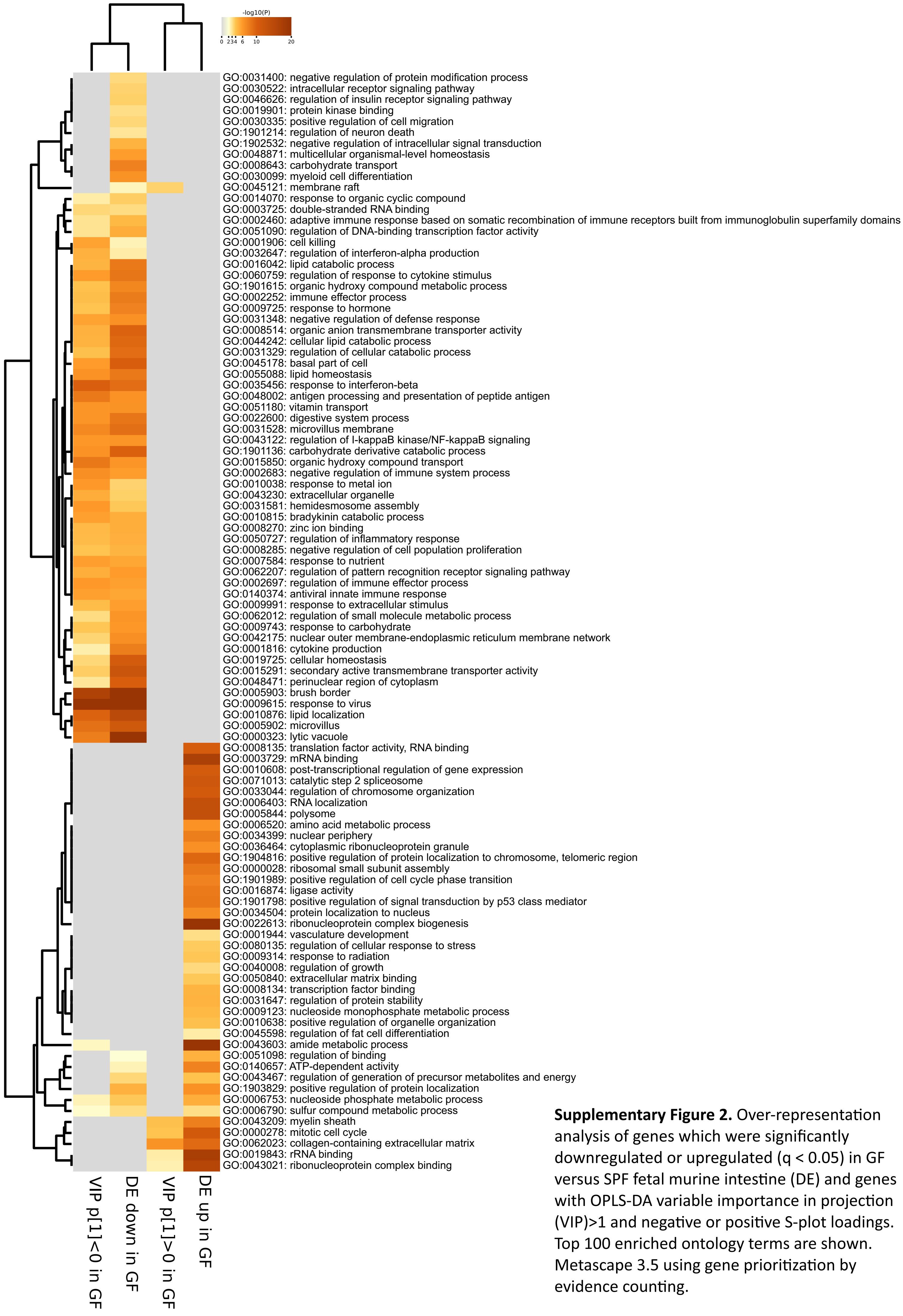

Supplementary Figure 2. Over-representation analysis of genes which were significantly downregulated or upregulated (q < 0.05) in GF versus SPF fetal murine intestine (DE) and genes with OPLS-DA variable importance in projection (VIP)>1 and negative or positive S-plot loadings. Top 100 enriched ontology terms are shown. Metascape 3.5 using gene prioritization by evidence counting.

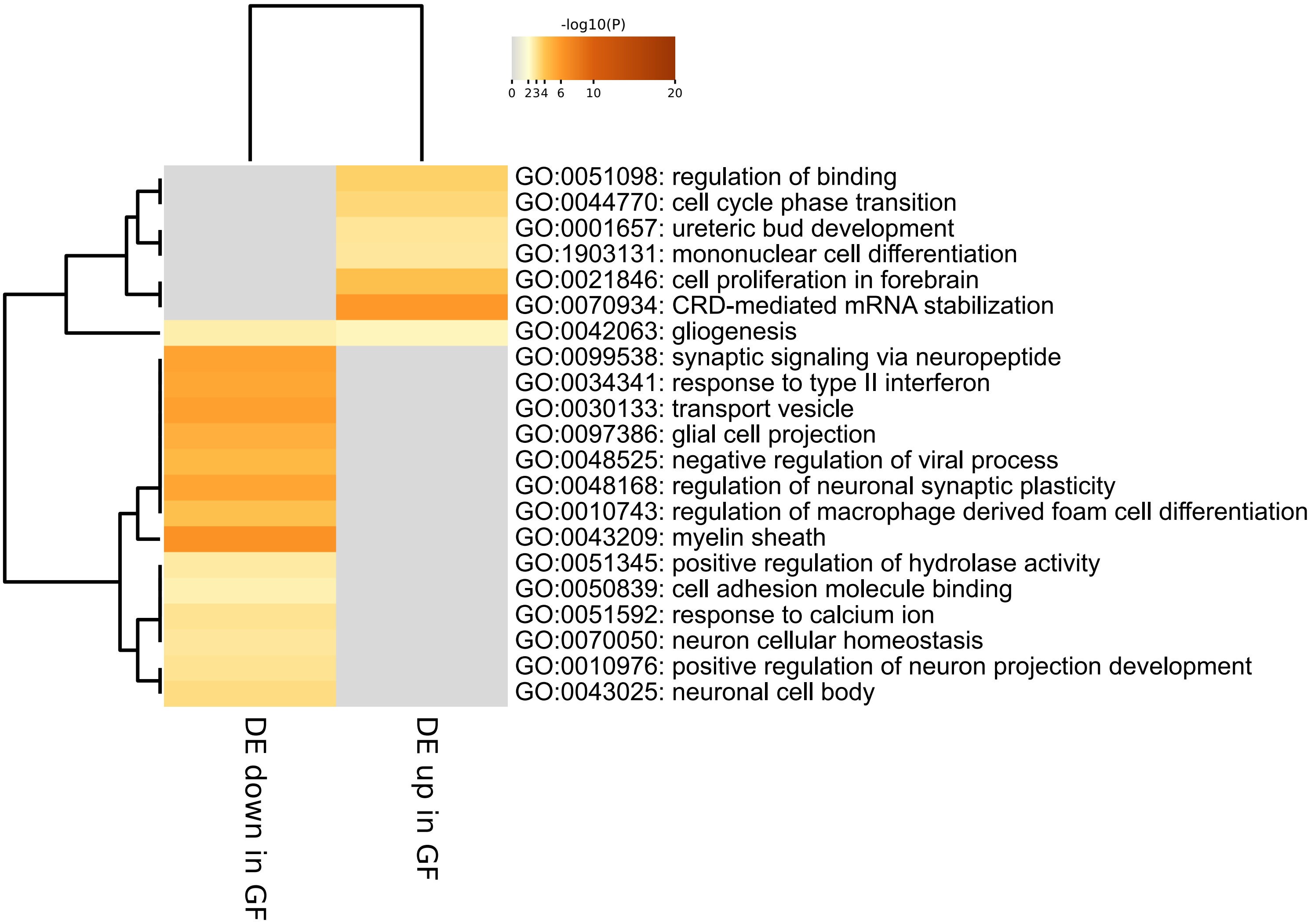

Supplementary Figure 3. Over-representation analysis of genes which were significantly downregulated or upregulated ($q < 0.05$) in GF versus SPF fetal murine brain.
Metascape 3.5 using gene prioritization by evidence counting.

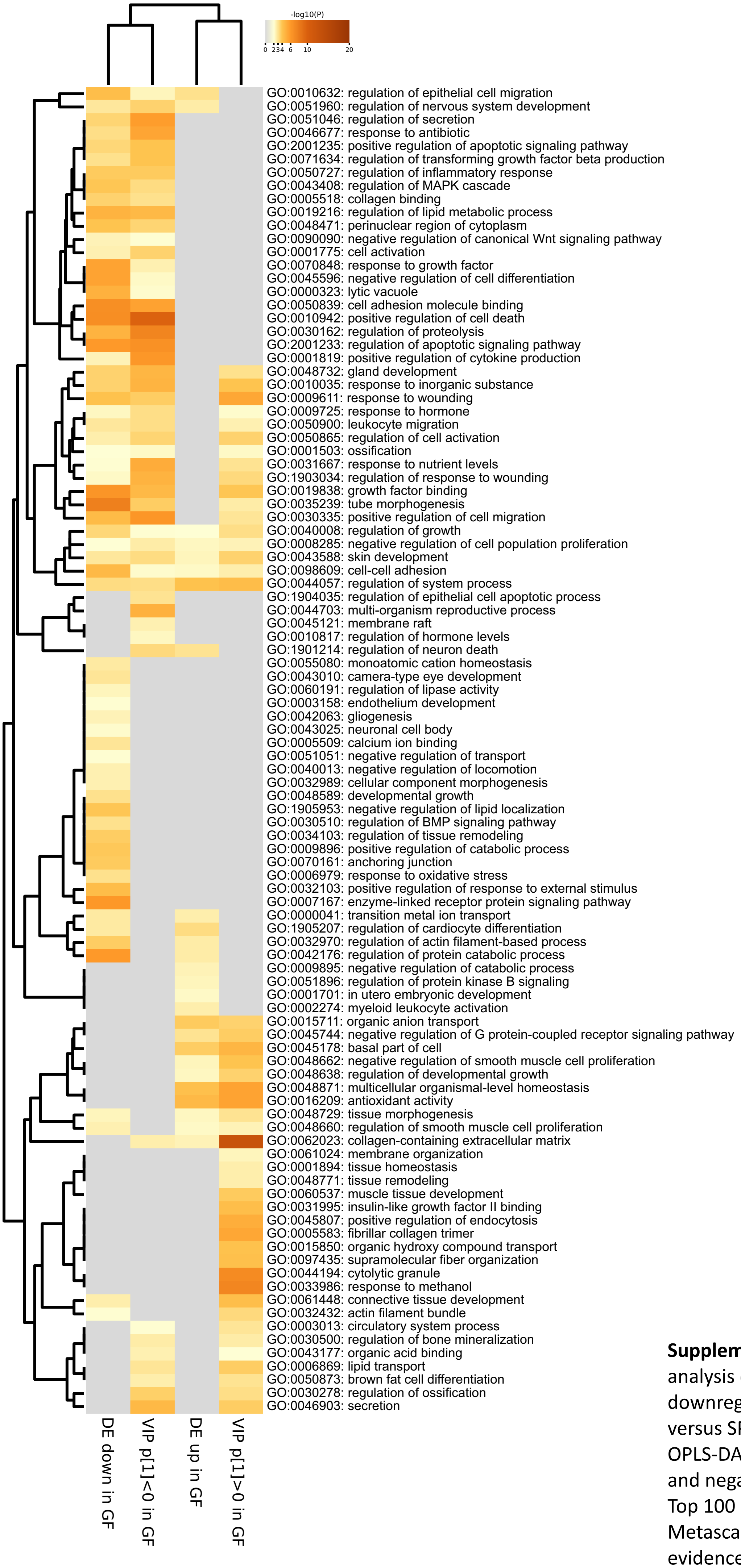

Supplementary Figure 4. Over-representation analysis of genes which were significantly downregulated or upregulated (q < 0.05) in GF versus SPF murine placenta (DE) and genes with OPLS-DA variable importance in projection (VIP)>1 and negative or positive S-plot loadings. Top 100 enriched ontology terms are shown. Metascape 3.5 using gene prioritization by evidence counting.

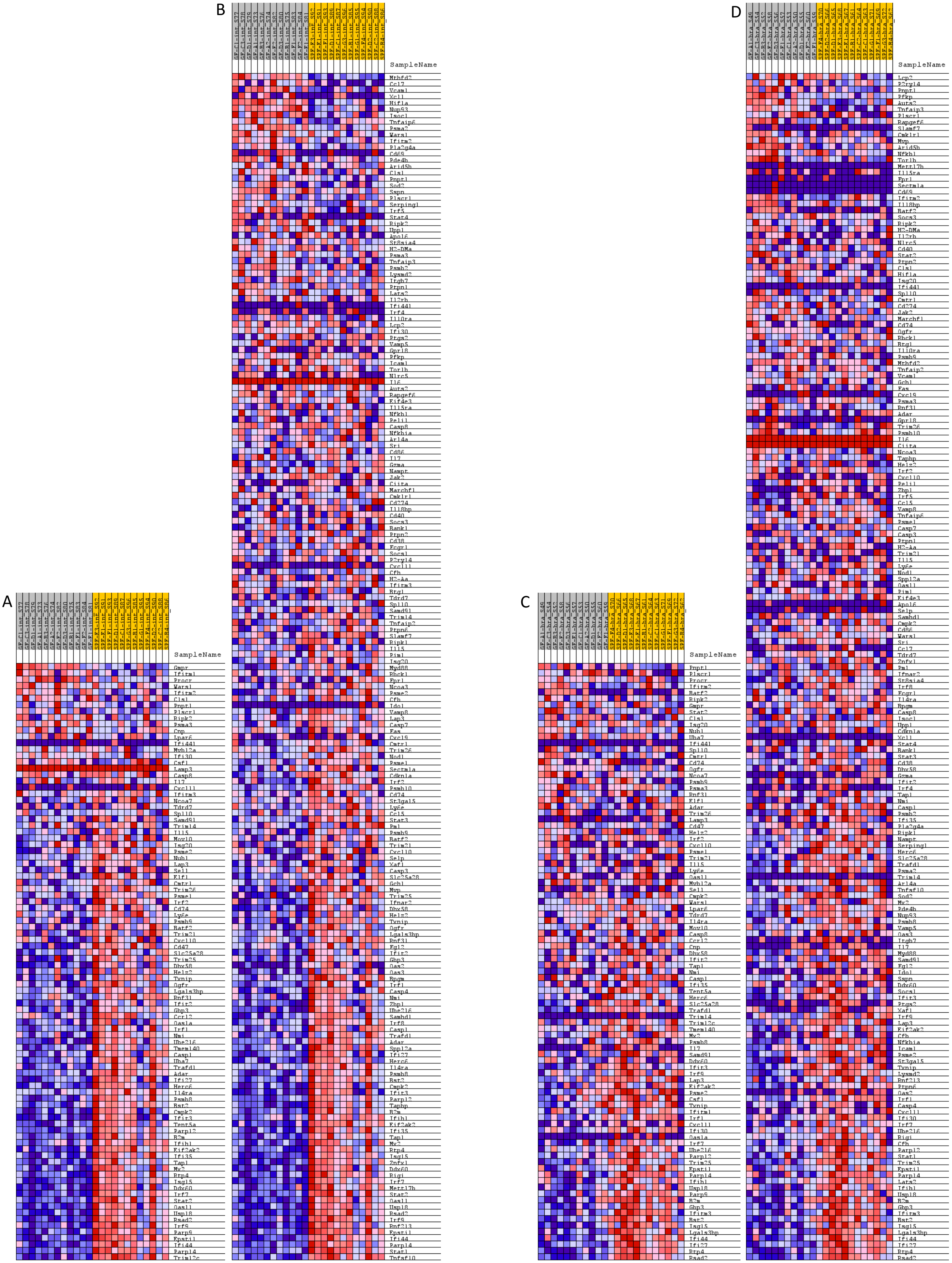

Supplementary Figure 5. Heatmaps for the interferon alpha and interferon gamma response gene sets.
a) Intestine Hallmark Interferon alpha Response, b) Intestine Hallmark Interferon gamma Response, c) Brain Hallmark Interferon alpha Response, d) Brain Hallmark Interferon gamma Response. Red = high expression, dark blue = lowest expression.

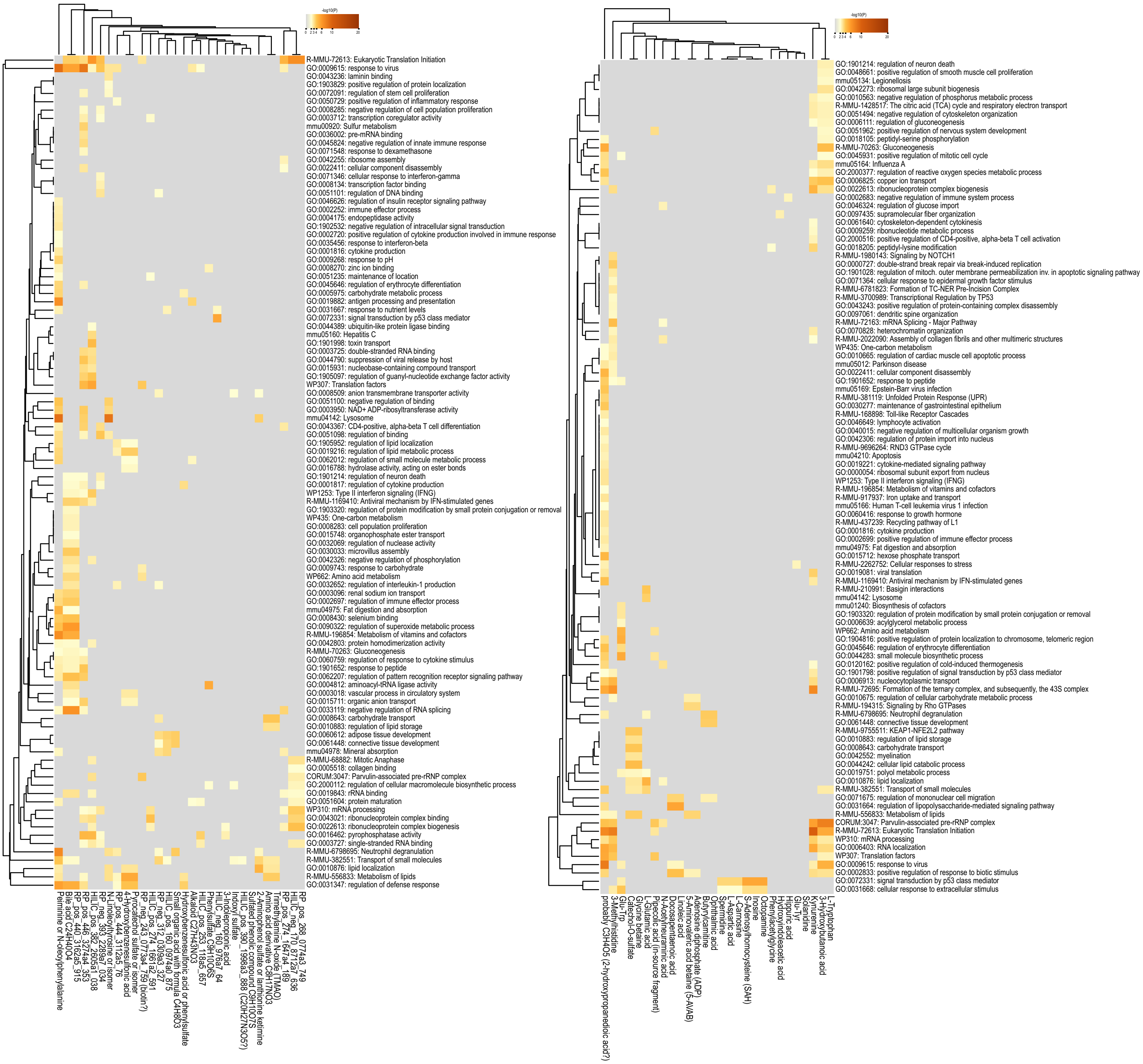

Supplementary Figure 6. Over-representation analysis of genes strongly associated with metabolites in fetal intestine. Highest-scoring molecular features missing from GF fetuses (left) and highest-scoring annotated metabolites more abundant in SPF fetuses (right). Top 100 enriched ontology terms are shown. Here, non-selective picking of ontology clusters was used.

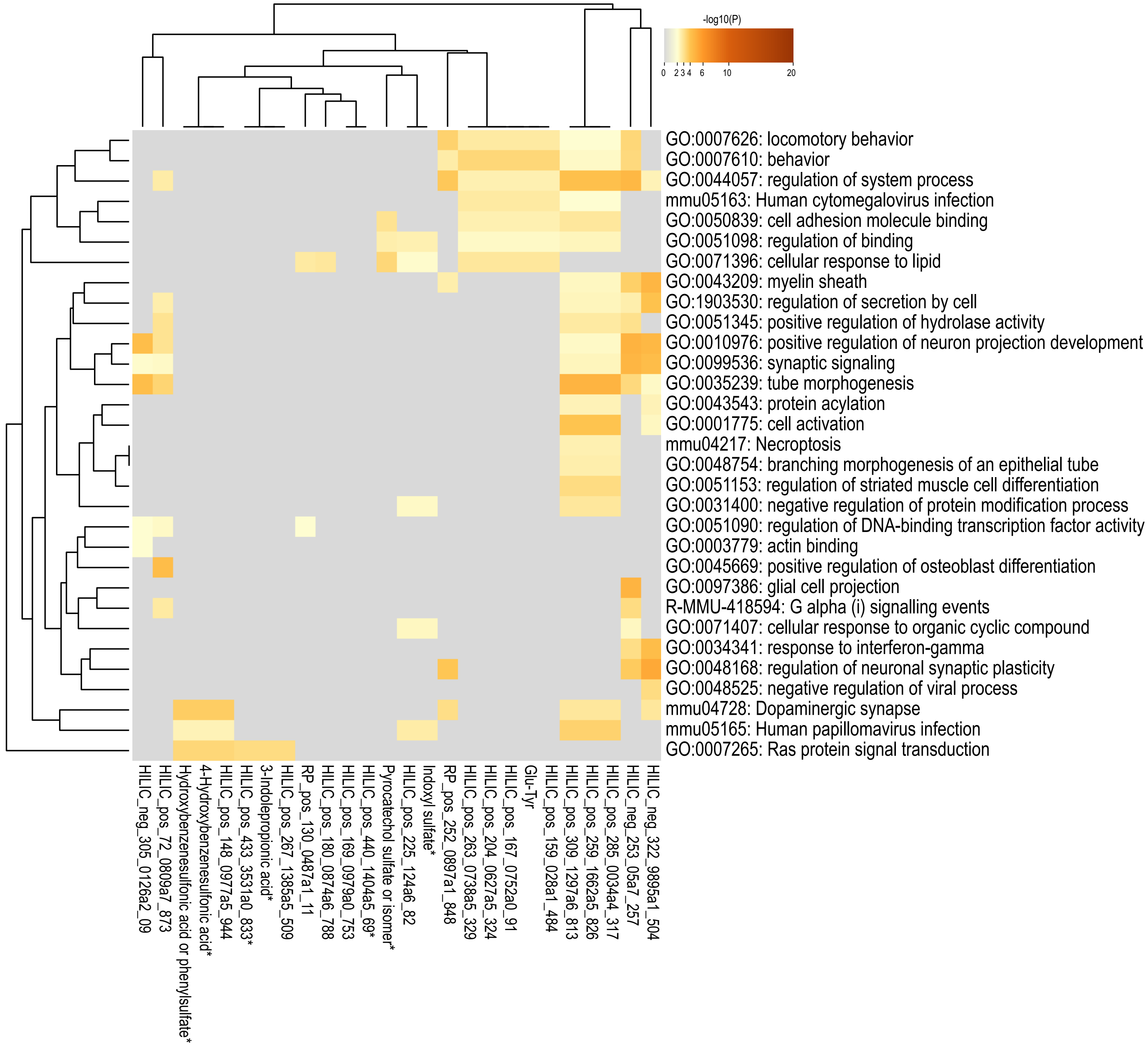

Supplementary Figure 7. Over-representation analysis of genes strongly associated with metabolites in fetal brain. Metabolites missing from GF fetuses are indicated by *. Top 100 enriched ontology terms are shown. Here, non-selective picking of ontology clusters was used.

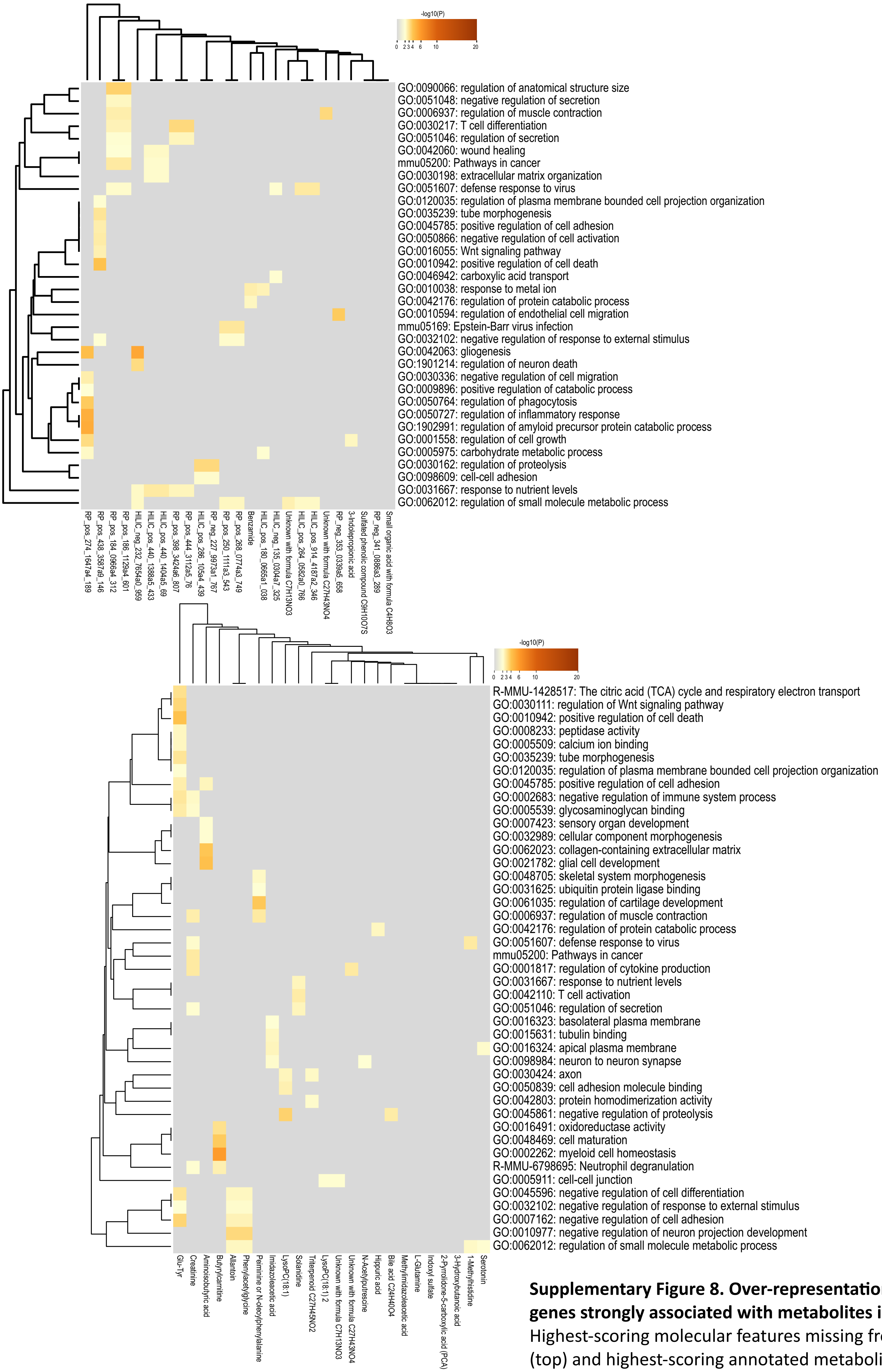

Supplementary Figure 8. Over-representation analysis of genes strongly associated with metabolites in placenta. Highest-scoring molecular features missing from GF fetuses (top) and highest-scoring annotated metabolites more abundant in SPF fetuses (bottom). Here, non-selective picking of ontology clusters was used.
